# Anti-Inflammatory Principles from the Needles of *Pinus taiwanensis* Hayata and In Silico Studies of Their Potential Anti-Aging Effects

**DOI:** 10.3390/antiox10040598

**Published:** 2021-04-13

**Authors:** Ping-Chung Kuo, Yue-Chiun Li, Anjar M. Kusuma, Jason T. C. Tzen, Tsong-Long Hwang, Guan-Hong Ye, Mei-Lin Yang, Sheng-Yang Wang

**Affiliations:** 1 School of Pharmacy, College of Medicine, National Cheng Kung University, Tainan 701, Taiwan; ycli0126@gmail.com (Y.-C.L.); anjarmahardian@gmail.com (A.M.K.); elva10352@gmail.com (G.-H.Y.); l3891104@nckualumni.org.tw (M.-L.Y.); 2 Graduate Institute of Biotechnology, National Chung-Hsing University, Taichung 402, Taiwan; tctzen@dragon.nchu.edu.tw; 3 Graduate Institute of Natural Products, College of Medicine, Chang Gung University, Taoyuan 333, Taiwan; htl@mail.cgu.edu.tw; 4 Research Center for Chinese Herbal Medicine, Research Center for Food and Cosmetic Safety, Graduate Institute of Health Industry Technology, College of Human Ecology, Chang Gung University of Science and Technology, Taoyuan 333, Taiwan; 5 Department of Anesthesiology, Chang Gung Memorial Hospital, Taoyuan 333, Taiwan; 6 Department of Forestry, National Chung-Hsing University, Taichung 402, Taiwan; taiwanfir@dragon.nchu.edu.tw

**Keywords:** *Pinus taiwanensis*, Pinaceae, anti-inflammatory, superoxide anion generation, elastase release, ghrelin receptor, molecular docking

## Abstract

*Pinus* needle tea are very popular in Eastern countries such as Japan, Russia, Korea, and China. Pine needle tea is claimed to have significant anti-aging effects, but no clear evidence has supported this until now. In the present study, five undescribed compounds (**1**–**5**) as well as seventy-two known compounds were purified and characterized from the bioactive fraction of methanol extracts of *P. taiwanensis* needles. Most of the isolates were examined for their anti-inflammatory bioactivity by cellular neutrophil model and six compounds (**45**, **47**, **48**, **49**, **50**, and **51**) exhibited a significant inhibition on superoxide anion generation and elastase release with IC_50_ values ranging from 3.3 ± 0.9 to 8.3 ± 0.8 μM. These anti-inflammatory ingredients were subjected to docking computing to evaluate their binding affinity on the ghrelin receptor, which played an important role in regulating metabolism, with anti-aging effects. Compounds **49**, **50**, and **51** formed a stable complex with the ghrelin receptor via hydrogen bonds and different types of interactions. These results suggest the flavonoids are responsible for the potential anti-aging effects of pine needle tea.

## 1. Introduction

*Pinus* genus (Pinaceae), comprising more than 100 species and are mainly distributed in the Northern Hemisphere, are generally evergreen trees while some are shrubs [1]. The pine wood is light and often used for furniture. However, various pharmacological effects of the needles on the pine shoots have been recorded in the ancient books of traditional Chinese medicine. In “The Divine Husbandman’s Herbal Foundation Canon”, pine needles promoted hair growth, prolonged life, and quenched thirst. In “Taiping Shenghui Fang”, it was recorded that pine needles could be cooked with alcohol and displayed an anti-aging effect. Nowadays, pine needles are processed as tea and are popular in Asia. In Korea, the constituents of the *P. densiflora* needle tea as well as its antioxidant and anti-bacterial bioactivities were extensively investigated [2,3]. Since early last century, more than seven hundred compounds have been identified from *Pinus* needles. Most of the reported literature are related to the essential oil of *P. densiflora* [4,5], *P. halepensis* [6,7], *P. nigra* [4,8], and *P. sylvestris* [4,9]. The characterized components of *Pinus* needles are mostly benzenoids, diterpenoids, flavonoids, lignans, monoterpenoids, and sesquiterpenoids [10]. The needles of *P. densiflora* and *P. morrisonicola* have been reported for their antioxidant bioactivity [11,12,13,14]. In addition, the ethyl acetate extract of needles of *P. morrisonicola* inhibited the protein and mRNA expression of NO and *i*NOS in LPS-induced RAW 264.7 macrophage, exhibiting the significant anti-inflammatory bioactivity [13]. The supercritical fluid extract of *P. densiflora* needles displayed the inhibitory effect on LPS-induced NO production by downregulating the expression of *i*NOS, and reduced the expression of IL-6 and IL-1β and activation of STAT1 and STAT3 proteins in macrophages induced by LPS [15]. The ethanol extract of *P. thunbergii* needles showed a significant anti-inflammatory effect in macrophages, and suppressed arachidonic acid-induced ear edema and inhibited myeloperoxidase enzymatic activity [16]. Moreover, the fermented *P. morrisonicola* needles showed their excellent antioxidant and anti-inflammatory bioactivities through modulating the NF-κB signaling pathway [17].

Ghrelin is a peptide hormone consisting of twenty-eight amino acids and was originally discovered in the stomach [18,19]. It is an endogenous ligand for the growth hormone secretagogue (GHSR), which is a member of the β-branch in class A GPCRs (G Protein-Coupled Receptors) [18,19]. Ghrelin is the only peptide hormone that causes hunger to promote appetite [20]. Many studies have suggested that ghrelin plays an important role in regulating metabolism, energy balance, memory, cardiovascular, and gastrointestinal functions in the human body [21,22,23]. Ghrelin is also involved in various physiological and pathophysiological mechanisms in the human body such as aging [24,25], and it may be related to anti-inflammatory activity [26,27]. In 2014, the unique acylated flavonoid tetraglycosides named teaghrelins, were first identified in Chin-shin oolong tea by our group and demonstrated their promoting activity in growth hormone (GH) release [28]. Two other similar compounds were purified in Shy-jih-chuen oolong tea and their bioactivity on the ghrelin receptor was also verified [29]. The four teaghrelins induced hunger through the same regulatory pathway as ghrelin. Not limited to tea, compounds with similar structures or bioactivities to teaghrelins have also been explored in *Polygonum multiflorum* (Heshouwu) [30], *Ginkgo biloba* [31], *Morus alba* [32], and *Cistanche tubulosa* [33]. These results support us in the search for natural anti-aging principles by screening the teaghrelin-like compounds that possess anti-inflammatory effects. In addition to directly performing the promoting activity in growth hormone release in cellular models, molecular modeling of some potential compounds would be more efficient in exploring possible candidates [34]. Computational methods have been applied to the development and evaluation of pharmacological hypothesis. Molecular docking is one of the most commonly utilized techniques and anticipates the conformation and affinity of ligand binding to the active pocket with high accuracy [35,36]. Docking methods effectively search high-dimensional spaces for possible interaction and use a scoring function that properly ranks the candidates [37].

In 2018, three compounds were purified from the needles of *P. morrisonicola* and exhibited significant vasorelaxant activity, and among these constituents, one was reported with a teaghrelin-like structure [38]. In Taiwan, *P. taiwanensis* needles are usually dried and baked and then processed as a tea due to their famous bioactivities. *P. taiwanensis* Hayata is one of the native and endemic species in Taiwan. It is also called Taiwan red pine, or huangshan pine. There are two needles in a fascicle with 8–11 cm long and slightly stiff and straight, and it grows across the whole island from low to high altitudes and often form pure forest [39]. In a preliminary examination, the ethyl acetate layer of methanol extracts of *P. taiwanensis* needles displayed a significant inhibition on superoxide anion generation and elastase release with IC_50_ values of 0.8 ± 0.2 and 1.0 ± 0.1 μg/mL, respectively (Table S1). Therefore, in the present study, the bioactive constituents of *P. taiwanensis* needles were investigated and the purified compounds were evaluated for their anti-inflammatory bioactivity on a cellular neutrophil model. In addition, the isolates from *P. taiwanensis* needles with anti-inflammatory bioactivities were subjected to docking computing and investigated for their interaction with the ghrelin receptor.

## 2. Materials and Methods

### 2.1. General Experimental Procedures

The melting points were recorded on a WRX-4 melting-point apparatus without correction. Optical rotations were recorded on a Jasco P-2000 digital polarimeter. The ultra violet (UV) spectra were obtained by a Hitachi U-2001 UV/V is spectrometer. The infrared (IR) spectra were examined with a Jasco FT/IR-4100 spectrophotometer. ^1^H-, ^13^C-, and 2D nuclear magnetic resonance (NMR) spectra were recorded on a Bruker AV-400 NMR spectrometer. Chemical shifts are shown in *δ* values (ppm) with tetramethylsilane as an internal standard. The *δ_H_* and *δ_C_* values were for the chemical shifts of the signals, respectively. High resolution electrospray ionization mass spectrometry (HR-ESI-MS) was conducted with a JEOL JMS-700 spectrometer (operated in the negative-ion mode).

### 2.2. Plant Material

The needles of *P. taiwanensis* were collected in Puli, Nantou, Taiwan and identified by Prof. Sheng-Yang Wang (Department of Forestry, National Chung-Hsing University, Taichung, Taiwan). The voucher specimen (PCKuo_2016003) was deposited in the herbarium of School of Pharmacy, National Cheng Kung University, Tainan, Taiwan.

### 2.3. Extraction and Isolation

The pine needles of *P. taiwanensis* (dried weight 2.5 kg) were powdered and extracted with methanol under reflux, the combined extracts were then concentrated in vacuo to obtain a brownish syrup (431 g). The methanol extract was partitioned between hexanes and water to remove the essential oil and produce the hexane layer (97 g) and water soluble. The water soluble part was further partitioned between ethyl acetate and water to yield the ethyl acetate layer (130 g) and water layer (204 g), respectively. The methanol extract, hexanes, ethyl acetate, and water layers were examined for their anti-inflammatory potential and only the ethyl acetate layer displayed a significant inhibition of superoxide anion generation and elastase release (see Supplementary Materials, Table S1). Therefore, the further isolation experiments were focused on this layer and the completed procedures are provided in the Supplementary Materials (Appendix A).

### 2.4. Spectral and Physical Data of 1–5

#### 2.4.1. 1-[(7′*R*,8′*S*)-7′,9′-Dihydroxy-7′-(4-hydroxyphenyl)propan-8′-yloxy]benzoic Acid (1)

Colorless syrup; [α]^25^_D_ + 5.1 (*c* 0.1, MeOH); UV (MeOH) *λ*_max_ (log *ε*) 248 (3.73), 228 (3.69) nm; ECD (*c* 3.65 × 10^−4^ M, MeOH) *λ*_max_ (Δ*ε*) 301 (+0.14), 230 (+0.17), 224 (+0.15) nm; IR (neat) *ν*_max_ 3412, 2925, 1598, 1550, 1390, 1242 cm^−1^; ^1^H and ^13^C NMR; HRESIMS *m/z* 303.0855 ([M − H]^−^ calcd for C_16_H_15_O_6_, 303.0869).

#### 2.4.2. 1-[(7′*R*,8′*S*)-7′,9′-Dihydroxy-7′-( 4-hydroxy-3-methoxyphenyl)propan-8′-yloxy]-2-hydroxybenzoic Acid (2)

Colorless syrup; [α]^25^_D_ + 5.3 (*c* 0.1, MeOH); UV (MeOH) *λ*_max_ (log *ε*) 284 (3.59), 253 (3.80) nm; ECD (*c* 3.51 × 10^−4^ M, MeOH) *λ*_max_ (Δ*ε*) 248 (+1.33), 224 (+1.03), 214 (+1.10) nm; IR (neat) *ν*_max_ 3425, 2927, 1541, 1384, 1271 cm^−1^; ^1^H and ^13^C NMR; HRESIMS *m/z* 349.0936 ([M − H]^−^ calcd for C_17_H_17_O_8_, 349.0923).

#### 2.4.3. (13*E*,12*R*)-12-Hydroxyagathic Acid (3)

Colorless powder; mp: 263 °C (dec.); [α]^25^_D_ + 30.8 (*c* 0.1, MeOH); UV (MeOH) *λ*_max_ (log *ε*) 225 (sh) (3.83) nm; IR (neat) *ν*_max_ 3450, 2937, 1648, 1252 cm^−1^; ^1^H-NMR (CD_3_OD, 400 MHz) *δ* 0.63 (3H, s, CH_3_-20), 1.09 (1H, ddd, *J* = 13.6, 13.6, 3.6 Hz, H-3a), 1.21 (3H, s, CH_3_-18), 1.21 (1H, m, H-1a), 1.42 (1H, m, H-5), 1.51 (1H, m, H-2a), 1.59 (2H, m, H-11), 1.79 (1H, m, H-1b), 1.91 (1H, m, H-2b), 1.91 (1H, m, H-6a), 1.98 (1H, m, H-7a), 2.01 (1H, m, H-6b), 2.08 (3H, s, CH_3_-16), 2.12 (1H, m, H-9), 2.14 (1H, m, H-3b), 2.43 (1H, m, H-7b), 4.03 (1H, dd, *J* = 9.2, 2.8 Hz, H-12), 4.53 (1H, s, H-17a), 4.91 (1H, s, H-17b), 5.88 (1H, br s, H-14); ^13^C-NMR (CD_3_OD, 100 MHz) *δ* 13.5 (CH_3_-20), 14.9 (CH_3_-16), 21.2 (C-2), 27.6 (C-6), 29.6 (CH_3_-18), 31.5 (C-11), 39.4 (C-3), 40.0 (C-7), 40.3 (C-1), 41.2 (C-10), 45.3 (C-4), 53.1 (C-9), 57.6 (C-5), 75.5 (C-12), 106.9 (C-17), 117.8 (C-14), 150.2 (C-8), 159.5 (C-13), 167.2 (C-15), 181.5 (C-19); HRESIMS *m/z* 349.2024 ([M − H]^−^ calcd for C_20_H_29_O_5_, 349.2015).

#### 2.4.4. 5-Isopropyl-3-oxocyclohex-1-ene-1-carboxylic Acid (4)

Colorless tabular crystal; mp: 235 °C (dec.); [α]^25^_D_ + 28.6 (*c* 0.3, MeOH); UV (MeOH) *λ*_max_ (log *ε*) 237 (sh) (3.40) nm; IR (neat) *ν*_max_ 3456, 2925, 1635, 1395 cm^-1^; ^1^H-NMR (CD_3_OD, 400 MHz) 0.88 (3H, d, *J* = 6.8 Hz, CH_3_-9), 0.98 (3H, d, *J* = 6.8 Hz, CH_3_-10), 1.84 (1H, m, H-4a), 2.02 (1H, m, H-4b), 2.09 (1H, m, H-5), 2.31 (1H, hept, *J* = 6.8 Hz, H-8), 2.50 (1H, dddd, *J* = 19.2, 9.2, 4.8, 2.4 Hz, H-6a), 2.70 (1H, dddd, *J* = 19.2, 5.2, 5.2, 1.2 Hz, H-6b), 6.32 (1H, dd, *J* = 2.4, 1.2 Hz, H-2); ^13^C-NMR (CD_3_OD, 100 MHz) *δ* 19.0 (C-9), 20.9 (C-10), 24.3 (C-4), 27.0 (C-6), 27.1 (C-8), 53.7 (C-5), 128.7 (C-2), 160.6 (C-1), 174.8 (C-7), 205.7 (C-3); HRESIMS *m/z* 181.0855 ([M − H]^−^ calcd for C_10_H_13_O_3_, 181.0865).

#### 2.4.5. Styraxinolic Acid (5)

Colorless syrup; UV (MeOH) *λ*_max_ (log *ε*) 308 (2.89), 237 (sh) (3.37), 222 (sh) (3.70) nm; IR (neat) *ν*_max_ 3421, 2926, 1572, 1395, 1268 cm^-1^; ^1^H-NMR (CD_3_OD, 400 MHz) 1.82 (2H, tt, *J* = 8.0, 6.8 Hz, H-8), 2.61 (2H, t, *J* = 8.0 Hz, H-7), 3.56 (2H, t, *J* = 6.8 Hz, H-9), 3.83 (3H, s, OCH_3_-3), 6.86 (1H, d, *J* = 2.4 Hz, H-4), 7.31 (1H, d, *J* = 2.4 Hz, H-6); ^13^C-NMR (CD_3_OD, 100 MHz) *δ* 32.7 (C-7), 35.6 (C-8), 56.6 (OCH_3_-3), 62.3 (C-9), 116.3 (C-4), 120.1 (C-1), 122.8 (C-6), 131.9 (C-5), 149.2 (C-3), 150.7 (C-2), 176.2 (C-10); HRESIMS *m/z* 225.0767 ([M − H]^−^ calcd for C_11_H_13_O_5_, 225.0763).

### 2.5. Anti-Inflammatory Bioactivity Examination

#### 2.5.1. Human Neutrophil Preparation

The study was conducted with the approval of the Institutional Review Board of Chang Gung Memorial Hospital (IRB No. 201800369A3). Blood samples were drawn from healthy human donors (20 to 30 years old), and neutrophils were isolated and purified according to the protocols described previously [40].

#### 2.5.2. Superoxide Anion Generation Measurement

The assay for measuring superoxide anion generation was based on the SOD-inhibitable reduction of ferricytochrome *c* as described previously [40].

#### 2.5.3. Elastase Release Assay

Degranulation of azurophilic granules was determined by measuring the release of elastase as previously described [40].

#### 2.5.4. Statistical Analysis

The results are expressed as mean ± standard error of the mean (SEM). Computation of 50% inhibitory concentrations (IC_50_) was performed using PHARM/PCS v.4.2 software. Statistical comparisons were made between groups using the Student’s *t*-test. Values of *p* < 0.05 were considered to be statistically significant.

### 2.6. Molecular Docking Study

The In Silico evaluation was conducted on AutoDock Vina software [41]. The crystal structure of the ghrelin receptor has been characterized [42], and a .PDB file was downloaded from the Protein Databank (PDB ID: 6KO5). The 3D structures of ligands were constructed in the Chem3D program. The hydrogen supplement, Gasteiger charge measurement for protein atoms, and selection of flexible torsions for ligands were conducted by AutodockTools (ADT ver. 1.5.6). The size of the grid was designed at 18.5 Å × 18.5 Å × 18.5 Å and a grid center at dimensions (x, y, and z, respectively): 9.7, –19.2, 14.6 was determined. The binding affinity energy was provided as docking scores and shown in kcal/mol. The best interaction was considered only the top-scoring pose. The visualization of the best docking interactions was analyzed in Biovia Discovery Studio client 2020 [43].

## 3. Results and Discussion

The pine needles were extracted with methanol and partitioned with hexanes, ethyl acetate, and water to obtain three soluble layers, respectively. The anti-inflammatory fraction, the ethyl acetate layer, was subjected to continuous conventional chromatographic technique combination, and five undescribed compounds were characterized including two new lignans, 1-[(7′*R*,8′*S*)-7′,9′-dihydroxy-7′-(4-hydroxyphenyl)propan-8′-yloxy]benzoic acid (**1**), 1-[(7′*R*,8′*S*)-7′,9′-dihydroxy-7′-(4-hydroxy-3-methoxyphenyl)-propan-8′-yloxy]-2-hydroxybenzoic acid (**2**), one new diterpenoid, (13*E*,12*R*)-12-hydroxyagathic acid (**3**), one monoterpenoid, 5-isopropyl-3-oxocyclohex-1-ene-1-carboxylic acid (**4**), and one phenylpropane, styraxinolic acid (**5**). The chemical structures of these new compounds were constructed with the assistance of the NMR spectral elucidation and MS spectrometric analysis. Moreover, seventy-two known compounds, comprising one steroid, *β*-sitosterol (**6**); one sesquiterpenoid, (‒)-oplopan-4-one-10-*α*-*O*-*β*-D-glucoside (**7**); one coumarin, umbelliferone (**8**); one alkaloid, indole-3-aldehyde (**9**); four diterpenoids, acrostalic acid (**10**), 15-hydroxy-7-oxo-8,11,13-abietatrien-18-oic acid (**11**), 3*β*,13-dihydroxylabda-8(20),14-dien-19-oic acid (**12**), 12,15-dihydroxylabda-8(17),13-dien-19-oic acid (**13**); twenty-six lignans, (2*S*,3*R*)-2,3-dihydro-3-hydroxymethyl-7-methoxy-2-(4′-hydroxy-3′-methoxyphenyl)-5-benzofuranpropanol 3*α*-*O*-*α*-L-rhamnopyranoside (**14**), (7*S*,8*R*)-dihydrodehydrodiconiferyl alcohol-9-*O*-*α*-L-rhamnopyranoside (**15**), icariside E_4_ (**16**), massonianoside B (**17**), (7*S*,8*R*)-dihydro-3′-hydroxy-8-hydroxymethyl-7-(4-hydroxy-3-methoxyphenyl)-1′-benzofuranpropanol (**18**), (±)-*rel*-(2*α*,3*β*)-7-*O*-methylcedrusin (**19**), cedrusinin (**20**), (7*S*,8*R*)-idaeusin D (**21**), (7*S*,8*R*)-4,9-dihydroxy-4′,7-epoxy-8′,9′-dinor-8,5′-neolignan-7′-oic acid (**22**), 2-[4-(3-hydroxypropyl)-2-methoxyphenoxy]propane-1,3-diol (**23**), evofolin-B (**24**), (*S*)-3-hydroxy-1,2-bis(4-hydroxy-3-methoxyphenyl)-1-propanone (**25**), cupressoside A (**26**), 1-(4′-hydroxy-3′-methoxyphenyl)-2-[2″-hydroxy-4″-(3-O-α-L-rhamnopyranosyloxypropyl)phenoxy]-1,3-propanediol (**27**), (7*R*,8*S*)-3-methoxy-8,4′-oxyneoligna-3′,4,7,9,9′-pentol (**28**), *erythro*-3-methoxy-8,4′-oxyneolignan-3′,4,7,9,9′-pentol (**29**), pinoresinol (**30**), (+)-salicifoliol (**31**), (+)-idaeusinol A (**32**), schizandriside (**33**), (+)-isolariciresinol 2*α*-*O*-*α*-L-arabinoside (**34**), (+)-isolariciresinol (**35**), secoisolariciresinol (**36**), secoisolariciresinol-9,9′-acetonide (**37**), (‒)-nortrachelogenin (**38**), (2*S*,3*S*)-2*α*-(4″-hydroxy-3″-methoxybenzyl)-3*β*-(4′-hydroxy-3′-methoxybenzyl)-*γ*-butyrolactone (**39**); twelve flavonoids, astragalin (**40**), kaempferol-3-*O*-*β*-D-galactopyranoside (**41**), kaempferol-3-*O*-*α*-L-furanoarabinoside (**42**), rhamnetin 3-*O*-*β*-D-glucopyranoside (**43**), apigenin (**44**), kaempferol-3,6-dimethyl ether (**45**), 5,7,8,4′-tetrahydroxy-3-methoxy-6-methylflavonol-8-*O*-*β*-D-glucopyranoside (**46**), 6-methylaromadendrin (**47**), naringenin (**48**), tiliroside (**49**), kaempferol 3-*O*-(3″,6″-di-*O*-*E*-*p*-coumaroyl)-*β*-D-glucopyranoside (**50**), kaempferol-3-*O*-(5″-*O*-*E*-*p*-coumaroyl)-*α*-L-arabinofuranoside (**51**); seven ionones, machilusoxide A (**52**), (+)-(*S*)-dehydrovomifoliol (**53**), isololiolide (**54**), (*S*)-(+)-abscisic acid sodium salt (**55**), (3*S*,5*R*,6*R*,7*E*)-3,5,6-trihydroxy-7-megastigmen-9-one (**56**), blumenol A (**57**), peltopterin B (**58**); nineteen benzenoids, 3,4-dihydroxybenzoic acid methyl ester (**59**), *p*-hydroxybenzoic acid (**60**), vanillic acid (**61**), 4-hydroxybenzaldehyde (**62**), methylparaben (**63**), 3-hydroxy-1-(4-hydroxy-3-methoxyphenyl)-1-propanone (**64**), 3-hydroxy-1-(4-hydroxyphenyl)-1-propanone (**65**), 2-(4-hydroxyphenyl)acetic acid (**66**), phenylacetic acid (**67**), isovanillic acid (**68**), benzoic acid (**69**), vanillin (**70**), *p*-hydroxyacetophenone (**71**), sodium salicylate (**72**), vannilic acid 4-*O*-*α*-L-rhamnoside (**73**), *trans*-ferulic acid (**74**), sodium *p*-coumarate (**75**), *p*-coumaric acid (**76**), *trans*-methyl *p*-coumarate (**77**), respectively, were identified by the examination of their physical and spectroscopic data with those previously published (references of known compounds were provided in Supplementary Materials Appendix B).

### 3.1. Structural Elucidation of Compounds 1–5

**Figure 1:**
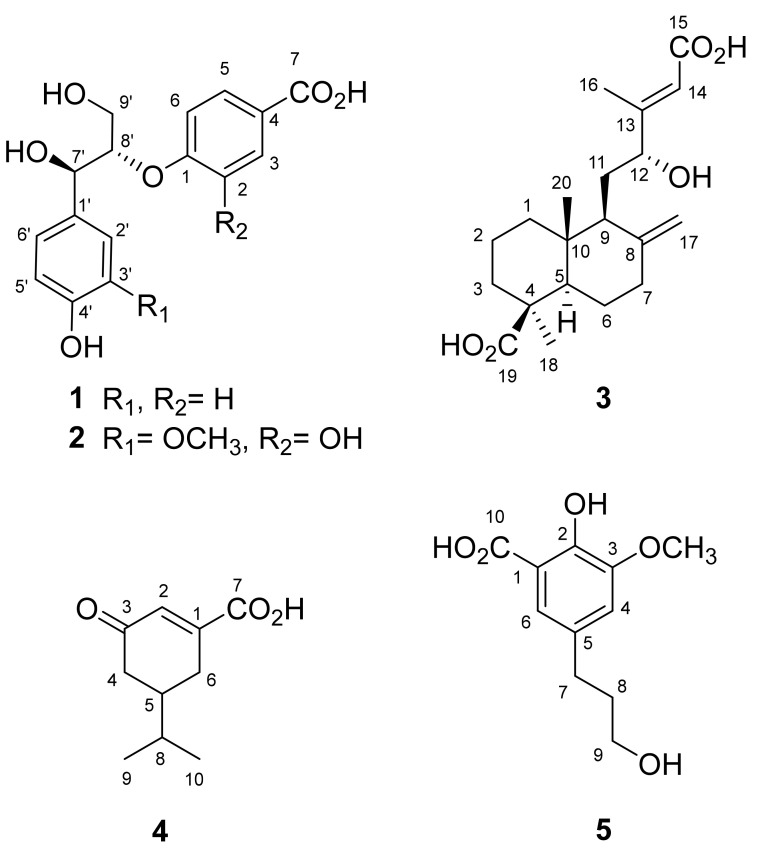
Structures of compounds **1**–**5** isolated from *P. taiwanensis*.

Compound **1** was isolated as an optically active colorless syrup, and the molecular formula was assigned as C_16_H_16_O_6_ by HR-ESI-MS analysis ([M − H]^−^, *m/z* 303.0855, calcd. for C_16_H_15_O_6_, 303.0869, Figure S1). The IR spectrum indicates the presences of a hydroxyl (3412 cm^−1^) and a conjugated carbonyl group (1598 cm^−1^). The ^1^H-NMR data (Figure S2) showed the signals for two *para*-substituted aromatic moieties [δ_H_ 6.72 (2H, d, *J* = 8.4 Hz, H-3′, -5′), 6.86 (2H, d, *J* = 8.8 Hz, H-2, -6), 7.24 (2H, d, *J* = 8.4 Hz, H-2′, -6′), and 7.83 (2H, d, *J* = 8.8 Hz, H-3, -5)], two oxygenated methines [δ_H_ 4.48 (1H, m, H-8′), and 4.85 (1H, d, *J* = 5.6 Hz, H-7′)], and two methines [δ_H_ 3.81 (1H, dd, *J* = 12.0, 4.0 Hz, H-9′a), and 3.86 (1H, dd, *J* = 12.0, 5.6 Hz, H-9′b)]. The ^13^C and DEPT NMR spectra (Figure S3) of **1** displayed sixteen carbons, corresponding to one methylene group, two oxygenated carbons, twelve aromatic carbons, and one conjugated carbonyl (Table 1). The *^2^J-* and *^3^J-* HMBC correlations from H-2, -6 to C-1 and 4; from H-3, 5 to C-1 and 7; from H-2′, 6′ to C-4′ and 7′; from H-7′ to C-8′ and 9′; and from H-8′ to C-1, respectively, were observed in the HMBC spectrum of **1** (Figure S4). Moreover, a large coupling constant between H-7′ and H-8′ (*J* = 5.6 Hz) supported the relative configuration of **1** at C-7′/C-8′ as *threo* [44]. The absolute configurations at C-7′ and C-8′ of **1** were determined by electronic circular dichroism (ECD) analysis. The positive Cotton effect at 230 nm (Δ*ε* + 0.17) revealed an 8*S* configuration for **1,** according to the published literature [37,38] and therefore 7′*R* was also determined. Other 2D spectra (Figure S5–S7) furnished the full assignment of proton and carbon signals. Accordingly, the structure of **1** was assigned as 1-[(7′*R*,8′*S*)-7′,9′-dihydroxy-7′-(4-hydroxyphenyl)propan-8′-yloxy]benzoic acid as shown in Figure 1.

The molecular formula of **2** was assigned as C_17_H_18_O_8_ on the basis of HR-ESI-MS analytical data (*m/z* 349.0936 [M − H]^−^, Figure S8). The absorption in the IR spectrum (3425 and 1541 cm^−1^) indicated the hydroxyl and conjugated carbonyl functionalities, respectively. Comparison of the NMR spectra of **1** and **2**, it could observe that they possessed different aromatic moieties. Two sets of ABX-coupled aromatic ring and one methoxy group could be detected in the ^1^H- (Figure S9) and ^13^C-NMR (Figure S10) data of **2** (Table 1), and it suggested that compound **2** possessed two trisubstituted rather than *para*-disubstituted aromatic moieties. The significant HMBC correlations (Figure S11) from H-3 to C-1, C-5 and C-7; from H-6 to C-4; from OCH_3_-3′ to C-3′; from H-6′ to C-4′ and C-7′; from H-7′ to C-1′, C-2′, C-8′ and 9′; from H-8′ to C-1, respectively, established that the structure of **2** was also a neolignan skeleton. Through combination of a large coupling constant (*J*_7,8_ = 5.2 Hz) and positive Cotton effect at 230 nm (Δ*ε* + 1.14), the absolute configuration of **2** was assigned as the *threo*- and (7′*R*,8′*S*)-form, the same as **1** [44,45]. Other 2D spectra (Figure S12–S14) furnished the full assignment of proton and carbon signals. These findings concluded the structure of **2** as 1-[(7′*R*,8′*S*)-7′,9′-dihydroxy-7′-(4-hydroxy-3-methoxyphenyl)propan-8′-yloxy]-2-hydroxybenzoic acid (Figure 1).

Compound **3** was obtained as a colorless powder and its molecular formula was assigned as C_20_H_30_O_5_ on the basis of HR-ESI-MS analytical data (*m/z* 349.2024 [M − H]^−^, Figure S15). Compound **3** showed absorption peaks at 3450 (OH), and 1648 (carboxylic acid) cm^−1^ in its IR spectrum. It was evidenced by the ^13^C-NMR spectrum (Figure S16) in which two carboxylic functionalities were observed at δ_C_ 167.2 (C-15) and 181.5 (C-19). In its ^1^H-NMR (Figure S17), the resonances at δ_H_ 4.03 (1H, dd, *J* = 9.2, 2.8 Hz, H-12) and δ_C_ 75.5 (C-12) indicated the presence of a secondary alcohol group. The terminal methylene group could be established due to the proton resonances at δ_H_ 4.53 (1H, s, H-17a) and 4.91 (1H, s, H-17b), and the carbon signals at δ_C_ 106.9 (C-17) and 150.2 (C-8), respectively. Two methyl groups at δ_H_ 0.63 (3H, s, CH_3_-20) and 1.21 (3H, s, CH_3_-18) were connected to the quarternary carbons (C-10 and C-4) evidenced by the HMBC correlations (Figure S18). The shielding effect of the carboxylic group at C-4 resulted in the upfield shift of CH_3_-20 (δ_H_ 0.63), suggesting its *β*-configuration [46]. In addition, the chemical shift of H-17a (δ_H_ 4.53) appeared in the upfield region, suggesting the 12*R* configuration [47]. In its HMBC spectrum, the correlations from H-12 to C-9, C-14 and C-16; from H-17 to C-7 and C-9; from CH_3_-16 to C-12 and C-14; from CH_3_-18 to C-3, C-4 and C-19; from CH_3_-20 to C-1, C-5, C-9 and C-10, respectively, constructed the planar structure of **3** as previously reported for 12-hydroxyagathic acid [46]. However, the C-13 configuration of **3** was determined as *E* by the NOESY analytical data (Figure S19), which displayed the NOE effects among H-5/H-9, H-5/H-18, and H-12/H-14. Moreover, the NOE between H-14 and CH_3_-16, which should be recorded in 12-hydroxyagathic acid [46], was not detected in **3**. Other 2D spectra (Figure S20–S21) furnished the full assignment of proton and carbon signals. Conclusively, the structure of **3** was established as (13*E*,12*R*)-12-hydroxyagathic acid as shown (Figure 1).

The HR-ESI-MS spectrum of **4** exhibited an [M − H]^−^ ion peak at *m/z* 181.0855 (Figure S22), consistent with the pseudomolecular formula of C_10_H_13_O_3_. The absorption peaks at 3456 and 1635 cm^-1^ in its IR spectrum displayed hydroxyl and conjugated carbonyl groups, respectively. Two methine protons at δ_H_ 2.09 (1H, m, H-5) and 6.32 (1H, dd, *J* = 2.4, 1.2 Hz, H-2), two methylene groups at δ_H_ 1.84 (1H, m, H-4a), 2.02 (1H, m, H-4b), 2.50 (1H, dddd, *J* = 19.2, 9.2, 4.8, 2.4 Hz, H-6a) and 2.70 (1H, dddd, *J* = 19.2, 5.2, 5.2, 1.2 Hz, H-6b), and one set of isopropyl protons at δ_H_ 0.88 (3H, d, *J* = 6.8 Hz, CH_3_-9), 0.98 (3H, d, *J* = 6.8 Hz, CH_3_-10) and 2.31 (1H, hept, *J* = 6.8 Hz, H-8) appeared in the ^1^H-NMR spectrum of **4** (Figure S23). In addition, one conjugated carbonyl carbon at δ_C_ 174.8 (C-7), and one carboxyl carbon at δ_C_ 205.7 (C-3) could be observed in its ^13^C- and DEPT NMR spectra (Figure S24). The observed HMBC correlations (Figure S25) from H-2 to C-7; from H-4 to C-3; from H-6 to C-1, C-2, and C-5; from CH_3_-9 to C-5, and CH_3_-10; from CH_3_-10 to C-8, respectively, constructed the structure of **4** as 5-isopropyl-3-oxocyclohex-1-ene-1-carboxylic acid (Figure 1). Other 2D spectra (Figure S26–S28) furnished the full assignment of proton and carbon signals. However, the stereochemistry at C-5 remained undetermined.

Compound **5** possessed the molecular formula C_11_H_14_O_5_ determined from a deprotonated molecular ion peak in the negative mode HR-ESI-MS analysis (*m/z* 225.0767 [M − H]^−^, Figure S29). In its IR spectrum, hydroxyl (3421 cm^−1^) and carboxyl (1572 cm^−1^) functionalities could be detected. The ^1^H-NMR spectrum (Figure S30) revealed two long-range coupling aromatic protons at δ_H_ 6.86 (1H, d, *J* = 2.4 Hz, H-4) and 7.31 (1H, d, *J* = 2.4 Hz, H-6), one methoxy group at δ_H_ 3.56 (2H, t, *J* = 6.8 Hz, H-9), and one set of propanol protons at δ_H_ 1.82 (2H, tt, *J* = 8.0, 6.8 Hz, H-8), 2.61 (2H, t, *J* = 8.0 Hz, H-7) and 3.56 (2H, t, *J* = 6.8 Hz, H-9). Moreover, one carboxylic group was located at δ_C_ 176.2 (C-10) in its ^13^C-NMR spectrum (Figure S31). The planar structure of **5** was established by the significant HMBC correlations (Figure S32) of OCH_3_-3 to C-3; H-6 to C-2, C-4, C-7, and C-10; H-7 to C-4, C-5, and C-8; H-9 to C-7, and C-8, respectively. Other 2D spectra (Figure S33–S35) furnished the full assignment of proton and carbon signals. The above evidence suggests the structure of **5** as 2-hydroxy-5-(3-hydroxypropyl)-3-methoxybenzoic acid (Figure 1), which was already reported as styraxinolic acid in the previous synthetic literature [48]. Nevertheless, the present research is the first report of **5** from natural sources.

### 3.2. Anti-Inflammatory Activity

**Figure 2:**
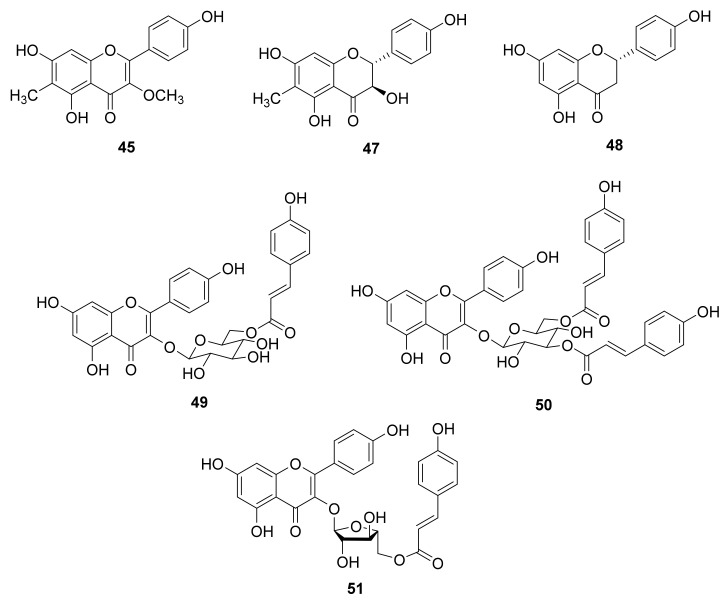
Structures of anti-inflammatory principles **45**, **47**, **48**, **49**, **50**, and **51**.

Inflammation is one of the major self-defense mechanisms stimulated by bacteria, virus, wound, or various other environmental factors. It is a first response of the immune system against infection and irritation. Neutrophils belong to an abundant kind of macrophage and play a major role in inflammation, and are usually the first lymphocytes to reach the infected region [49]. Neutrophils secrete a series of cytotoxins such as superoxide anion and elastase in response to the activation of the immune system [50]. In recent years, various human diseases have been demonstrated to be related to neutrophil overexpression [51,52,53,54,55]. The relationship between inflammation and cancer has been established, and the authors pointed out that the formation of cancer cells was directly related to inflammation [49]. Therefore, new anti-inflammatory compounds are worthwhile for further study on cancer treatment. Forty-three isolated compounds were evaluated for the inhibition of superoxide anion generation and elastase release by human neutrophils in response to fMLF/CB [56] (see Supplementary Materials, Table S2). The significant inhibitory results (Table 2) demonstrated that only **45**, **47**, **48**, **49**, and **50** (Figure 2) displayed a significant inhibition of superoxide anion generation, with IC_50_ values ranging from 3.3 ± 0.9 to 7.7 ± 0.9 μM compared with the positive control LY294002 (IC_50_ 1.1 ± 0.3 μM). Moreover, **48**, **50**, and **51** (Figure 2) revealed the significant inhibition of elastase release with IC_50_ values ranging from 5.3 ± 0.2 to 8.3 ± 0.8 μM compared with the positive control LY294002 (IC_50_ 3.2 ± 1.0 μM) (Table 2). Compounds **48** and **50** displayed both inhibition of superoxide anion generation and elastase release, indicating their multiple anti-inflammatory bioactivities. The needles of *P. morrisonicola* have been reported to have an anti-inflammatory effect in RAW 264.7 macrophages [13]. The authors proposed that epicatechin and *p*-coumaric acid identified in *P. morrisonicola* may be the active ingredients. In the present research, all the active compounds contained the flavone backbone similar to that of epicatechin and the *p*-coumaroyl moiety could also be observed in **49**, **50**, and **51**. This indicates that the flavonoid and *p*-coumaroyl functional groups may contribute the anti-inflammatory bioactivity in the present study. These bioassay results suggest that flavonoids play key roles in *Pinus* species for anti-inflammation bioactivity.

### 3.3. Molecular Docking Study

The age-related decline in GH levels is considered to be a symptom of neuroendocrine aging [57]. This phenomenon exists in several mammalian species such as humans, domestic dogs, and laboratory rodents [57]. In human, the GH levels in plasma begins to decrease with age after full physical maturation, and continues during the decades of life [57]. Ghrelin is identified as the endogenous ligand for the GHSR and is a main regulator of GH secretion [18,19]. Ghrelin is involved in various physiological and pathophysiological mechanisms in humans such as aging [24,25]. In addition, ghrelin may be thought to be related to anti-inflammatory activity. Immune cell activation was limited by ghrelin treatment through the inhibition of NF-κB activation and subsequent MCP-1 secretion [26]. A synthetic ghrelin analog growth hormone-releasing peptide-2 (GHRP-2) was reported to reduce the inflammatory factors in arthritic rats, and it supports that the immune cells were mediated by the activation of ghrelin receptors [27]. Neves et al. also proposed the regulation of inflammation as an anti-aging intervention [58]. Thus, according to the anti-inflammatory bioassay experimental data, **45**, **47**, **48**, **49**, **50**, and **51** (Figure 2) showed significant inhibitory effects, and were selected to determine their binding abilities to the ghrelin receptor. Before docking simulation, the native ligand (8QX) included in the 6KO5 PDB file was re-docked for validation. The interactions between 8QX and 6KO5 and the best pose of calculated results showed high similarity and repeatability with native data (data not shown). The results indicate the high accuracy of the existing simulation system and supported the further computing. 

**Figure 3:**
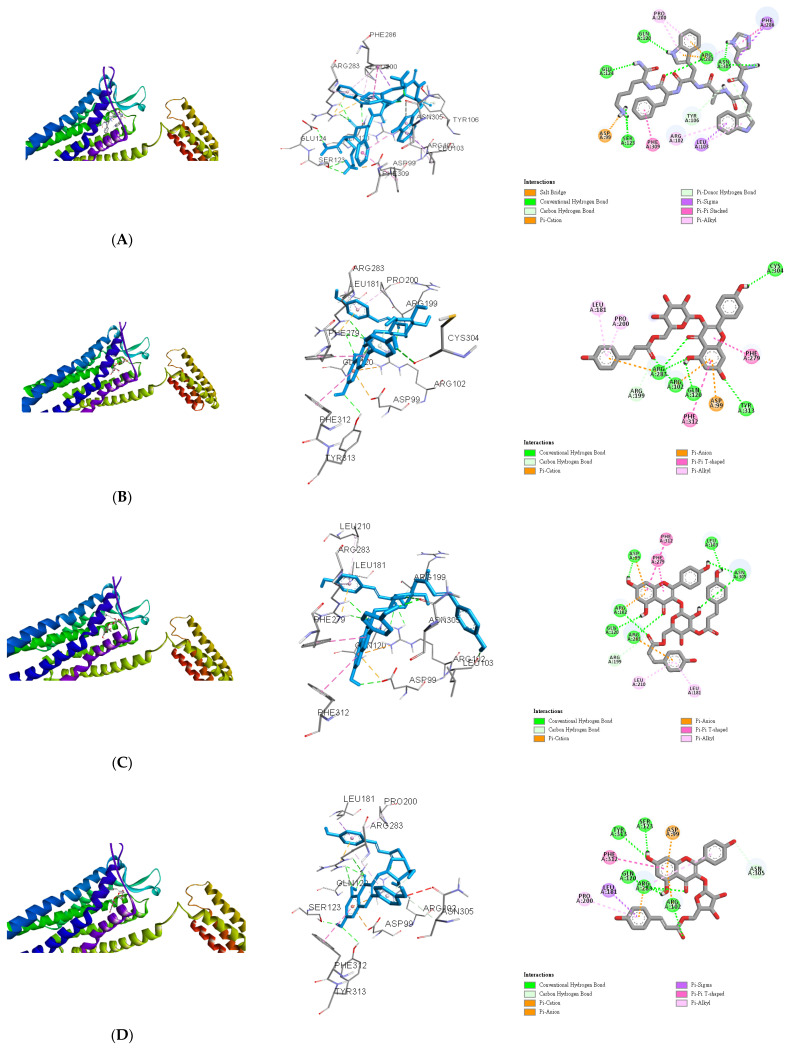
In Silico modeling of (**A**) GHRP-6, (**B**) **49**, (**C**) **50**, and (**D**) **51** docking into the ghrelin receptor.

The lowest binding energy of each ligand was considered the best conformation. The binding affinities are listed in Table 3. Growth hormone-releasing peptide 6 (GHRP-6) was used as a positive control for docking to the binding pocket of the ghrelin receptor as in our previous report. Although AutoDock Vina is not constructed for docking between peptides and proteins, several successful results have been published in previous reports [59,60,61]. Therefore, in this study, GHRP-6 was first computed to determine the accuracy of the present docking model and the results coincided well (Figure 3A). Compared with GHRP-6, the binding energies of **49**, **50**, and **51** were lower than −10.3 kcal/mol (Table 3). This suggests that **49**, **50**, and **51** could dock into the pocket of the ghrelin receptor similar or even better than that of GHRP-6. For **49**, the hydrogen bonds could be observed between two carbonyl groups (C-4 and *p*-coumaroyl) and Arg283, C-5 hydroxyl and Gln120, C-7 hydroxyl and Tyr313, and C-4′ hydroxyl and Cys304, respectively. Arg199 formed a conventional hydrogen bond with a carbonyl group of *p*-coumaroyl. In addition, **49** was linked to the Arg283, Arg102, Asp99, Phe279, Phe312, Leu181, and Pro200 residues of the ghrelin receptor via different effects such as the π-cation, π-anion, π–π T-shaped, and π-alkyl interactions. These allowed compound **49** and protein to form a stable complex (Figure 3B). **50** was bound with Asp99, Arg102, Gln120, Arg283, Leu103, Asn305, and Arg199 through various hydrogen bonds, while other interactions (π–cation, π–anion, π–π T-shaped, and π–alkyl) were also observed with Asp99, Arg102, Arg283, Phe279, Phe312, Leu181, and Leu210 (Figure 3C). **51** also established hydrogen or carbon hydrogen bonds with Tyr313, Ser123, Arg283, Arg102 and Asn305, together with other interactions (π-cation, π-anion, π-sigma, π–π T-shaped, and π-alkyl) to link with Asp99, Arg283, Arg102, Leu181, Phe312, and Pro200 residues of ghrelin receptor could be detected (Figure 3D). Compared compounds **45**, **47**, and **48** with **49**, **50**, and **51**, the former group possessed a flavonoid skeleton only while the latter group had sugar and coumaroyl moieties. It was reported that the coumaroyl group attached on the sugar was crucial for the binding affinity to the ghrelin receptor [34]. The gap structure of GHSR interacting with the acyl acid moiety of ghrelin resulted in the transformation of the ghrelin receptor into an active configuration [43]. Moreover, the binding pocket of GHSR is bifurcated by the salt bridge between Glu124 and Arg283, and this region is rich in hydrophobic amino acids [43]. According to our data, the docking scores of **49**, **50**, and **51** were higher than those of **45**, **47**, and **48**, which suggested the better binding capability. The major structural characteristics were the coumaroyl groups rather than the sugar moieties and this could be further evidenced by examination of more compounds possessing coumaroyl functionalities. In this study, the active ingredients **49**, **50**, and **51** possessed not only anti-inflammatory bioactivity, but also the ghrelin receptor binding potential. This indicated that the claimed anti-aging effects of pine needle tea may be also based on these teaghrelin-like compounds. 

## 4. Conclusions

A total of seventy-seven isolates comprising five undescribed compounds were purified from the methanol extracts of *P. taiwanensis* needles. Their structures were characterized through spectroscopic and spectrometric analyses. Forty-three purified compounds were examined for their anti-inflammatory activity by the inhibition of superoxide anion generation and elastase release on neutrophil model. The results suggest that **45**, **47**, **48**, **49**, **50**, and **51** possess significant anti-inflammatory potentials. Further molecular docking computing results supported **49**, **50**, and **51** exhibiting a binding affinity to the active pocket of the ghrelin receptor. Therefore, the crude extracts and purified constituents of *P. taiwanensis* have the potential to be developed as new anti-inflammatory lead drugs or food ingredients.

## Figures and Tables

**Table 1:** ^1^H and ^13^C NMR spectroscopic data of compounds **1** and **2**.

Position	1		2	
	*δ*_H_	*δ*c	*δ*_H_	*δ*c
1	–	162.1	–	150.2
2	6.86 (2H, d, *J* = 8.8 Hz)	116.3	–	148.1
3	7.83 (2H, d, *J* = 8.8 Hz)	132.0	7.45 (1H, d, *J* = 2.0 Hz)	118.3
4	–	116.1	–	132.4
5	7.83 (2H, d, *J* = 8.8 Hz)	132.0	7.38 (1H, dd, *J* = 8.4, 2.0 Hz)	122.5
6	6.86 (2H, d, *J* = 8.8 Hz)	116.3	6.96 (1H, d, *J* = 8.4 Hz)	116.5
7	–	175.4	–	174.4
1′	–	133.5	–	134.0
2′	7.24 (2H, d, *J* = 8.4 Hz)	129.2	7.02 (1H, d, *J* = 2.0 Hz)	111.4
3′	6.72 (2H, d, *J* = 8.4 Hz)	115.9	–	148.9
4′	–	157.9	–	147.2
5′	6.72 (2H, d, *J* = 8.4 Hz)	115.9	6.74 (1H, d, *J* = 8.4 Hz)	115.9
6′	7.24 (2H, d, *J* = 8.4 Hz)	129.2	6.84 (1H, dd, *J* = 8.4, 2.0 Hz)	120.6
7′	4.85 (1H, d, *J* = 5.6 Hz)	73.8	4.93 (1H, d, *J* = 5.2 Hz)	73.8
8′	4.48 (1H, m)	84.1	4.33 (1H, m)	86.3
9′	3.81 (1H, dd, *J* = 12.0, 4.0 Hz) 3.86 (1H, dd, *J* = 12.0, 5.6 Hz)	62.0	3.55 (1H, dd, *J* = 12.0, 5.2 Hz) 3.78 (1H, dd, *J* = 12.0, 4.4 Hz)	61.7
OCH_3_-3′	–	–	3.81 (3H, s)	56.3

^1^H- and ^13^C-NMR data (*δ* in ppm) were measured in CD_3_OD at 400 and 100 MHz. “s”, “d”, “m”, and “dd” were for the singlet, doublet, multiplet, and doublet of doublet signals, respectively.

**Table 2:** Inhibitory effects of purified compounds on superoxide anion generation and elastase release by human neutrophils in response to fMLF/CB.

Compound	Superoxide Anion Generation		Elastase Release	
	IC_50_ (μM) ^a^	Inh % ^b^	IC_50_ (μM)	Inh %
**45**	6.4 ± 0.7	70.5 ± 6.8 ***	– ^c^	34.2 ± 6.9 **
**47**	6.0 ± 1.1	71.8 ± 8.1 ***	–	43.5 ± 6.9 ***
**48**	3.3 ± 0.9	87.5 ± 5.4 ***	5.3 ± 0.2	93.9 ± 5.2 ***
**49**	7.7 ± 0.9	60.6 ± 3.9 ***	–	40.3 ± 6.0 **
**50**	5.3 ± 1.1	72.9 ± 6.3 ***	5.8 ± 0.9	81.4 ± 12.0 ***
**51**	–	45.2 ± 5.6 ***	8.3 ± 0.8	57.0 ± 4.6 ***
LY294002 ^d^	1.1 ± 0.3	100.6 ± 1.0 ***	3.2 ± 1.0	76.7 ± 6.8 ***

Results are presented as mean ± SEM (*n* = 3-5). ** *p* < 0.01, *** *p* < 0.001 compared with the control (DMSO). ^a^ Concentration necessary for 50% inhibition (IC_50_). ^b^ Percentage of inhibition (Inh %) at 10 μM concentration. ^c^ Not determined. ^d^ A phosphatidylinositol-3-kinase inhibitor was used as a positive control.

**Table 3:** Binding energies of compound **45–51**, and GHRP-6 calculated In Silico.

Compound	Affinity (kcal/mol)
**45**	−8.8
**47**	−8.2
**48**	−8.2
**49**	−10.5
**50**	−11.0
**51**	−10.7
GHRP-6	−10.3

## Data Availability

Original data can be obtained from corresponding author upon request.

## Supplementary Materials

The following are available online at https://www.mdpi.com/article/10.3390/antiox10040598/s1, Appendix A: Complete extraction and isolation procedures, Appendix B: References of known compounds, Table S1: Preliminary bioactivity screening of needles of *P. taiwanensis* on superoxide anion generation and elastase release by human neutrophils in response to fMLF/CB, Table S2: Inhibitory effects of purified compounds on superoxide anion generation and elastase release by human neutrophils in response to fMLF/CB, Figure S1–S35: HRMS and NMR spectra of new compounds **1**–**5**.
