# Supplementary material for: Anti-Inflammatory Principles from the Needles of Pinus taiwanensis Hayata and In Silico Studies of Their Potential Anti-Aging Effects
Source: Antioxidants (Basel). 2021 Apr 13;10(4):598. doi: 10.3390/antiox10040598 (PMC8069155; doi:10.3390/antiox10040598)

Supplementary Materials

Anti-Inflammatory Principles from the Needles of *Pinus taiwanensis* Hayata and *In Silico* Studies of Their Potential Anti-Aging Effects

Ping-Chung Kuo [1,*], Yue-Chiun Li [1,a], Anjar M. Kusuma [1,a], Jason T. C. Tzen [2], Tsong-Long Hwang [3,4,5], Guan-Hong Ye [1], Mei-Lin Yang [1], and Sheng-Yang Wang [6]

[1] School of Pharmacy, College of Medicine, National Cheng Kung University, Tainan 701, Taiwan; z10502016@ncku.edu.tw (P.-C.K.); ycli0126@gmail.com (Y.-C.L.); anjarmahardian@gmail.com (A.M.K.); elva10352@gmail.com (G.-H.Y.); l3891104@nckualumni.org.tw (M.-L.Y.)

[2] Graduate Institute of Biotechnology, National Chung-Hsing University, Taichung 402, Taiwan; tctzen@dragon.nchu.edu.tw

[3] Graduate Institute of Natural Products, College of Medicine, Chang Gung University, Taoyuan 333, Taiwan; htl@mail.cgu.edu.tw

[4] Research Center for Chinese Herbal Medicine, Research Center for Food and Cosmetic Safety, Graduate Institute of Health Industry Technology, College of Human Ecology, Chang Gung University of Science and Technology, Taoyuan 333, Taiwan;

[5] Department of Anesthesiology, Chang Gung Memorial Hospital, Taoyuan 333, Taiwan;

[6] Department of Forestry, National Chung-Hsing University, Taichung 402, Taiwan; taiwanfir@dragon.nchu.edu.tw

* Correspondence: z10502016@ncku.edu.tw; Tel.: +886-6-2353535 # 6806

[a] These authors contributed equally to this work

ORCID

Ping-Chung Kuo: 0000-0003-3019-1138

Yue-Chiun Li: 0000-0002-1706-1139

Jason T.C. Tzen: 0000-0002-4218-6363

Contents

Appendix A. Complete extraction and isolation procedures.

Appendix B. References of the known compounds.

Table S1. Preliminary bioactivity screening of needles of *P. taiwanensis* on superoxide anion generation and elastase release by human neutrophils in response to fMLF/CB.

Table S2. Inhibitory effects of purified compounds on superoxide anion generation and elastase release by human neutrophils in response to fMLF/CB.

Figure S1. High resolution electrospray ionization mass spectrometry (HR-ESI-MS) spectrum of **1**.

Figure S2. ^{1}H nuclear magnetic resonance (NMR) spectrum of **1** (CD_3OD, 400 MHz).

Figure S3. ^{13}C and distortionless enhancement by polarization transfer (DEPT) NMR spectrum of **1** (CD_3OD, 100 MHz).

Figure S4. Heteronuclear multiple bond correlation (HMBC) spectrum of **1** (CD_3OD, 400 MHz).

Figure S5. Nuclear Overhauser effect spectroscopy (NOESY) spectrum of **1** (CD_3OD, 400 MHz).

Figure S6. Correlation spectroscopy (COSY) spectrum of **1** (CD_3OD, 400 MHz).

Figure S7. Heteronuclear single quantum correlation (HSQC) spectrum of **1** (CD_3OD, 400 MHz).

Figure S8. HR-ESI-MS spectrum of **2**.

Figure S9. ^{1}H NMR spectrum of **2** (CD_3OD, 400 MHz).

Figure S10. ^{13}C and DEPT NMR spectrum of **2** (CD_3OD, 100 MHz).

Figure S11. HMBC spectrum of **2** (CD_3OD, 400 MHz).

Figure S12. NOESY spectrum of **2** (CD_3OD, 400 MHz).

Figure S13. COSY spectrum of **2** (CD_3OD, 400 MHz).

Figure S14. HSQC spectrum of **2** (CD_3OD, 400 MHz).

Figure S15. HR-ESI-MS spectrum of **3**.

Figure S16. ^{13}C and DEPT NMR spectrum of **3** (CD_3OD, 100 MHz).

Figure S17. ^{1}H NMR spectrum of **3** (CD_3OD, 400 MHz).

Figure S18. HMBC spectrum of **3** (CD_3OD, 400 MHz).

Figure S19. NOESY spectrum of **3** (CD_3OD, 400 MHz).

Figure S20. COSY spectrum of **3** (CD_3OD, 400 MHz).

Figure S21. HSQC spectrum of **3** (CD_3OD, 400 MHz).

Figure S22. HR-ESI-MS spectrum of **4**.

Figure S23. ^{1}H NMR spectrum of **4** (CD_3OD, 400 MHz).

Figure S24. ^{13}C and DEPT NMR spectrum of **4** (CD_3OD, 100 MHz).

Figure S25. HMBC spectrum of **4** (CD_3OD, 400 MHz).

Figure S26. NOESY spectrum of **4** (CD_3OD, 400 MHz).

Figure S27. COSY spectrum of **4** (CD_3OD, 400 MHz).

Figure S28. HMQC spectrum of **4** (CD_3OD, 400 MHz).

Figure S29. HR-ESI-MS spectrum of **5**.

Figure S30. ^1H NMR spectrum of **5** (CD_3OD, 400 MHz).

Figure S31. ^{13}C and DEPT NMR spectrum of **5** (CD_3OD, 100 MHz).

Figure S32. HMBC spectrum of **5** (CD_3OD, 400 MHz).

Figure S33. NOESY spectrum of **5** (CD_3OD, 400 MHz).

Figure S34. COSY spectrum of **5** (CD_3OD, 400 MHz).

Figure S35. HSQC spectrum of **5** (CD_3OD, 400 MHz).

Appendix A. Complete Extraction and Isolation Procedures

The pine needles of *P. taiwanensis* (dried weight 2.5 kg) were powdered and extracted with methanol under reflux, and the combined extracts were then concentrated in vacuo to obtain a brownish syrup (431 g). The methanol extract was resolved with hexanes and water to remove essential oil and produce a hexane layer (97 g) and water soluble. The water soluble was further partitioned between ethyl acetate and water to yield ethyl acetate layer (130 g) and water layer (204 g), respectively.

The ethyl acetate layer was resolved on a reverse-phase Diaion HP-20 column and eluted with a step gradient mixture of water and methanol (10:0, 7:3, 5:5, 3:7, 0:10) to afford 20 fractions (PTE 1–20). The fourth fraction (PTE 4) was recrystallized and gave **59** (5 mg). The eighth fraction was further column chromatographed on a Diaion HP-20 with a mixture of water and methanol (step gradient from 10:0 to 0:10) to afford nine subfractions (PTE 8.1–8.9). The fourth subfraction (PTE 8.4) was purified by preparative thin layer chromatography (pTLC) eluted with a solvent mixture of chloroform, methanol, and water (15:1:0.1) to yield **60** (6 mg). The fifth subfraction (PTE 8.5) was further isolated by Sephadex LH-20 column chromatography with a gradient mixture of water and methanol (10:0 to 0:10) to produce eleven minor fractions (PTE 8.5.1–8.5.11) The minor fraction (PTE 8.5.1) was applied to pTLC with a solvent mixture of chloroform and methanol (50:1) to give **53** (2 mg) and **56** (1 mg). The third minor fraction (PTE 8.5.3) was purified by repeated column chromatography over silica gel eluted with a step gradient mixture of chloroform and methanol (30:1 to 1:1) to result in **23**, (5 mg), **29** (4 mg), **52** (1 mg), **54** (2 mg), **57** (2 mg), and **58** (1 mg). The fifth minor fraction (PTE 8.5.5) was resolved on a silica gel column eluted with chloroform and a step gradient of methanol (50:1 to 1:1) to afford ten minor subfractions (PTE 8.5.5.1–8.5.5.10). Compound **5** (1.6 mg), **62** (6 mg), **64** (2 mg), and **65** (1 mg) were obtained from 8.5.5.3 by repeated silica gel column chromatography with chloroform and methanol eluent (50:1 to 1:1). The seventh minor subfraction (PTE 8.5.5.7) was purified by silica gel column chromatography with a mixture of chloroform and methanol (20:1) and further recrystallization of the resulting fractions afforded **61** (10 mg), **66** (3 mg), and **73** (4 mg). The minor fraction (PTE 8.5.9) was subjected to pTLC eluted with a solvent mixture of chloroform and acetone (1:2) to yield **1** (2 mg) and **2** (2 mg). The eleventh fraction was isolated on a reverse-phase Diaion HP-20 column and eluted with a step gradient solvent mixture of water and methanol (10:0 to 0:10) to produce six subfractions (PTE 11.1–11.6). The second subfraction (PTE 11.2) was further purified by Sephadex LH-20 eluted with water and methanol (10:0 to 0:10) to obtain eleven minor fractions. Minor fraction (PTE 11.2.3) was subjected to pTLC eluted with a solvent mixture of chloroform and methanol (6:1) to give **72** (3 mg). Compound **17** (3 mg), **28** (4 mg), and **33** (4 mg) were yielded from 11.2.7 by pTLC eluted with a solvent

mixture of chloroform and methanol (10:1). The eighth minor fraction (PTE 11.2.8) was purified by pTLC eluted with a solvent mixture of chloroform and methanol (10:1) to give **15** (3 mg), **34** (2 mg), and **75** (6 mg). A solvent mixture of chloroform and methanol (15:1) was used for 11.2.10 purification, then compound **18** (2 mg) and **35** (6 mg) were provided. The fifth subfraction (PTE 11.5) was resolved on a Sephadex LH-20 column eluted with a solvent mixture of water and methanol (10:0 to 0:10) to give sixteen minor fractions (PTE 11.5.1–11.5.16). Compound **7** (1 mg), **27** (2 mg), **31** (4 mg), **32** (3 mg), **55** (2 mg), and **67** (3 mg) were produced from 11.5.2 by repeated Sephadex LH-20 column chromatography with a water and methanol eluent (10:0 to 0:10). The fifth minor fraction (PTE 11.5.5) was subjected to pTLC eluted with a solvent mixture of chloroform and methanol (50:1) to yield **14** (2 mg), **16** (5 mg), **70** (8 mg), and **71** (3 mg). Minor fraction 11.5.7 was purified by silica gel column chromatography eluted with a step gradient of chloroform and acetone (50:1 to 1:1) to afford ten minor subfractions (PTE 11.5.7.1–11.5.7.10). The second minor subfraction (PTE 11.5.7.2) was separated by pTLC with a solvent mixture of chloroform and acetone (20:1) to afford **8** (3 mg) and **63** (5 mg). The minor subfraction 11.5.7.3 was recrystallized from ethyl acetate to give **68** (7 mg). Compounds **36** (4 mg) and **37** (2 mg) were obtained from 11.5.7.5 by pTLC eluted with a solvent mixture of chloroform and acetone (20:1). The eighth minor fraction (PTE 11.5.8) was resolved with silica gel column chromatography eluted with a step gradient of chloroform and acetone (20:1 to 1:1) to give **9** (1 mg), **19** (2 mg), and **24** (3 mg). A repeated column chromatography eluted with chloroform and acetone (step gradient from 50:1 to 1:1) resulted in **21** (1 mg), **25** (2 mg), **38** (2 mg), **76** (5 mg), and **77** (8 mg). The thirteenth minor fraction (PTE 11.5.13) was isolated by pTLC with chloroform and methanol (6:1) to provide **22** (2 mg), **40** (2 mg), and **43** (1 mg). The fourteenth minor fraction (PTE 11.5.14) was subjected to pTLC eluted with a solvent mixture of chloroform and ethyl acetate (1:1) to produce **41** (2 mg) and **42** (1 mg). The fifteenth fraction (PTE 15) was separated by Diaion HP-20 column chromatography with a solvent mixture of water and methanol (step gradient from 10:0 to 0:10) to generate eight subfractions (PTE 15.1–15.8). The third subfraction (PTE 15.3) was further resolved on a MCI gel CHP20P eluted with water and methanol (10:0 to 0:10) to afford fifteen minor fractions (PTE 15.3.1–15.3.15). Compound **4** (3 mg) and **69** (12 mg) were given from 15.3.7 by pTLC with a solvent mixture of chloroform and methanol (5:1). The fourth subfraction (PTE 15.4) was divided into thirteen minor fractions (PTE 15.4.1–15.4.13) by MCI gel CHP20P column chromatography eluted with water and methanol (step gradient from 10:0 to 0:10). A repeated silica gel column chromatography was performed from 15.4.11 eluted with a step gradient of chloroform and methanol (50:1 to 1:1) and compounds **20** (2 mg), **26** (2 mg), and **47** (4 mg) were obtained. A recrystallization was completed and resulted in compound **74** (8 mg) from 15.6. Subfraction 15.7 was purified by Sephadex LH-20 gel column chromatography eluted with water and methanol

(10:0 to 0:10) to afford sixteen minor fractions (PTE 15.7.1–15.7.16). The sixth minor fraction (PTE 15.7.6) was further isolated by silica gel column chromatography with chloroform and methanol (step gradient from 50:1 to 1:1) to give nine minor subfractions (PTE 15.7.6.1–15.7.6.9). The fourth minor subfraction (PTE 15.7.6.4) was separated by pTLC eluted with a solvent mixture of benzene and acetone (30:1) to give **12** (2 mg) and **39** (2 mg). Minor subfraction 15.7.6.7 was purified by repeated column chromatography over silica gel eluted with a step gradient mixture of chloroform and acetone (30:1 to 1:1) to yield **3** (1.4 mg), **10** (2 mg), and **13** (3 mg). A separation was executed from 15.7.8 eluted with a solvent mixture of chloroform and acetone (10:1) to get **46** (1 mg). The eleventh minor fraction (PTE 15.7.11) was separated by pTLC eluted with a solvent mixture of chloroform and methanol (8:1) to produce **48** (2 mg) and **51** (2 mg). The seventeenth fraction (PTE 17) was resolved and loaded into a Sephadex LH-20 column chromatography eluted with water and methanol (10:0 to 0:10) to obtain eight subfractions (PTE 17.1–17.8). The same procedure was performed on 17.3 to give eleven minor fractions (PTE 17.3.1–17.3.11). The fifth minor fraction (PTE 17.3.5) was isolated by a repeated silica gel column chromatography eluted with a step gradient of chloroform and acetone (50:1 to 1:1) to generate **6** (5 mg), **11** (2 mg), and **30** (3 mg). The subfraction 17.7 was purified by pTLC with a solvent mixture of chloroform and acetone (10:1) to obtain **44** (1 mg) and **45** (2 mg). An Amberlite XAD-7 gel column chromatography eluted with water and methanol (10:0 to 0:10) was executed from 17.8 and resulted in twelve minor fractions (PTE 17.8.1–17.8.12). Two separated procedures were performed from 17.8.7 including pTLC with a solvent mixture of chloroform and methanol (6:1), and recrystallization led to **49** (2 mg) and **50** (1 mg).

Appendix B. References of Known Compounds

β-sitosterol (**6**): Kuo, Y.H.; Li, Y.C. Constituents of the bark of *Ficus microcarpa* Lf. *J. Chin. Chem. Soc.* **1997**, *44*, 321–325. https://doi.org/10.1002/jccs.199700048

(–)-oplopan-4-one-10-*α*-*O*-*β*-D-glucoside (**7**): Lee, K.H.; Choi, S.U.; Lee, K.R. Sesquiterpenes from *Syneilesis palmata* and their cytotoxicity against human cancer cell linesin *vitro*. *Arch. Pharmacal. Res.* **2005**, *28*, 280–284. https://doi.org/10.1007/BF02977792

umbelliferone (**8**): Li, H.; Bos, A.; Jean, S.; Webster, D.; Robichaud, G.A.; Johnson, J.A.; Gray, C.A. Antimycobacterial 1,4-napthoquinone natural products from *Moneses uniflora*. *Phytochem. Lett.* **2018**, *27*, 229–233. https://doi.org/10.1016/j.phytol.2018.07.032

indole-3-aldehyde (**9**): Zhao, C.; Ying, Z.; Tao, X.; Jiang, M.; Ying, X.; Yang, G. A new lactam alkaloid from *Portulaca oleracea* L. and its cytotoxity. *Nat. Prod. Res.* **2018**, *32*, 1548–1553. https://doi.org/10.1080/14786419.2017.1385022

acrostalic acid (**10**): Yuan, L.; Zhao, P.J.; Ma, J.; Lu, C.H.; Shen, Y.M. Labdane and tetranorlabdane diterpenoids from *Botryosphaeria* sp. MHF, an endophytic fungus of *Maytenus hookeri*. *Helv. Chim. Acta* **2009**, *92*, 1118–1125. https://doi.org/10.1002/hlca.200800424

15-hydroxy-7-oxo-8,11,13-abietatrien-18-oic acid (**11**): Yang, X.W.; Feng, L.; Li, S.M.; Liu, X.H.; Li, Y.L.; Wu, L.; Shen, Y.H.; Tian, J.M.; Zhang, X.; Liu, X.R.; et al. Isolation, structure, and bioactivities of abiesadines A-Y, 25 new diterpenes from *Abies georgei* Orr. *Bioorg. Med. Chem.* **2010**, *18*, 744–754. https://doi.org/10.1016/j.bmc.2009.11.055

3*β*,13-dihydroxylabda-8(20),14-dien-19-oic acid (**12**): Rodrigues-Filho, E.; Magnani, R.F.; Xie, W.; Mirocha, C.J.; Pathre, S.V. Hydroxylation of the labdane diterpene cupressic acid by *Fusarium graminearum*. *J. Braz. Chem. Soc.* **2002**, *13*, 266–269. https://doi.org/10.1590/S0103-50532002000200020

12,15-dihydroxylabda-8(17),13-dien-19-oic acid (**13**): Wang, Y.Z.; Tang, C.P.; Ke, C.Q.; Weiss, H.C.; Gesing, E.R.; Ye, Y. Diterpenoids from the pericarp of *Platycladus orientalis*. *Phytochemistry* **2008**, *69*, 518–526. https://doi.org/10.1016/j.phytochem.2007.07.023

(2*S*,3*R*)-2,3-dihydro-3-hydroxymethyl-7-methoxy-2-(4'-hydroxy-3'-methoxyphenyl)-5-benzofuranpropanol 3*α*-*O*-*α*-L-rhamnopyranoside (**14**): Iida, N.; Inatomi, Y.; Murata, H.; Murata, J.; Lang, F.A.; Tanaka, T.; Nakanishi, T.; Inada, A. New phenylpropanoid glycosides from *Juniperus communis* var. *depressa*. *Chem. Pharm. Bull.* **2010**, *58*, 742–746. https://doi.org/10.1248/cpb.58.742

(7*S*,8*R*)-dihydrodehydrodiconiferyl alcohol-9-*O*-*α*-L-rhamnopyranoside (**15**): Nakanishi, T.; Iida, N.;

Inatomi, Y.; Murata, H.; Inada, A.; Murata, J.; Lang, F.A.; Iinuma, M.; Tanaka, T. Neolignan and flavonoid glycosides in *Juniperus communis* var. *depressa*. *Phytochemistry* **2004**, *65*, 207–213. https://doi.org/10.1016/j.phytochem.2003.10.025

icariside E_4 (**16**): Nakanishi, T.; Iida, N.; Inatomi, Y.; Murata, H.; Inada, A.; Murata, J.; Lang, F.A.; Iinuma, M.; Tanaka, T. Neolignan and flavonoid glycosides in *Juniperus communis* var. *depressa*. *Phytochemistry* **2004**, *65*, 207–213. https://doi.org/10.1016/j.phytochem.2003.10.025

massonianoside B (**17**): Chen, J.; Park, H.J. Computer-aided discovery of massonianoside B as a novel selective DOT1L inhibitor. *ACS Chem. Biol.* **2019**, *14*, 873–881. https://doi.org/10.1021/acschembio.8b00933

(7*S*,8*R*)-dihydro-3'-hydroxy-8-hydroxymethyl-7-(4-hydroxy-3-methoxyphenyl)-1'-benzofuranpropanol (**18**): Kim, T.H.; Ito, H.; Hayashi, K.; Hasegawa, T.; Machiguchi, T.; Yoshida, T. Aromatic constituents from the heartwood of *Santalum album* L. *Chem. Pharm. Bull.* **2005**, *53*, 641–644. https://doi.org/10.1248/cpb.53.641

(±)-*rel*-(2*α*,3*β*)-7-*O*-methylcedrusin (**19**): Li, J.L.; Li, N.; Xing, S.S.; Zhang, N.; Li, B.B.; Chen, J.G.; Ahn, J.S.; Cui, L. New neo-lignan from *Acanthopanax senticosus* with protein tyrosine phosphatase 1B inhibitory activity. *Arch. Pharm. Res.* **2017**, *40*, 1265–1270. https://doi.org/10.1007/s12272-015-0659-7

cedrusinin (**20**): Agrawal, P.K.; Rastogi, R.P. Two lignans from *Cedrus deodara*. *Phytochemistry* **1982**, *21*, 1459–1461. https://doi.org/10.1016/0031-9422(82)80172-6

(7*S*,8*R*)-idaeusin D (**21**): Zhou, L.; Xi, Y.F.; Wang, W.; Lin, B.; Wang, X.B.; Huang, X.X.; Song, S.J. Chiral resolution and bioactivity of enantiomeric benzofuran neolignans from the fruit of *Rubus ideaus* L. *Fitoterapia* **2018**, *127*, 56–61. https://doi.org/10.1016/j.fitote.2018.01.012

(7*S*,8*R*)-4,9-dihydroxy-4',7-epoxy-8',9'-dinor-8,5'-neolignan-7'-oic acid (**22**): Wang, Y.H.; Sun, Q.Y.; Yang, F.M.; Long, C.L.; Zhao, F.W.; Tang, G.H.; Niu, H.M.; Wang, H.; Huang, Q.Q.; Xu, J.J.; et al. Neolignans and caffeoyl derivatives from *Selaginella moellendorffii*. *Helv. Chim. Acta* **2010**, *93*, 2467–2477. https://doi.org/10.1002/hlca.201000116

2-[4-(3-hydroxypropyl)-2-methoxyphenoxy]propane-1,3-diol (**23**): Duan, W.; Pan, S.; Yu, Z.; Wang, X.; Li, J.; Zhang, Y. Studies on chemical constituents of twigs of *Trichosanthes kirilowii* Maxim. *Asian J. Chem.* **2015**, *27*, 2756–2758. https://doi.org/10.14233/ajchem.2015.18021

evofolin-B (**24**): Wu, T.S.; Yeh, J.H.; Wu, P.L. The heartwood constituents of *Tetradium glabrifolium*. *Phytochemistry* **1995**, *40*, 121–124. https://doi.org/10.1016/0031-9422(95)00248-6

(*S*)-3-hydroxy-1,2-bis(4-hydroxy-3-methoxyphenyl)-1-propanone (**25**): Woo, K.W.; Suh, W.S.; Subedi, L.; Kim, S.Y.; Choi, S.U.; Kim, K.H.; Lee, K.R. Phenolic derivatives from the stems of

Lagerstroemia indica and their biological activity. *Heterocycles* **2015**, *91*, 2355–2366. http://dx.doi.org/10.3987/COM-15-13328

cupressoside A (**26**): Xu, J.F.; Cao, D.H.; Tan, N.H.; Liu, Z.L.; Zhang, Y.M.; Yang, Y.B. New lignan glycosides from *Cupressus duclouxian* (Cupessaceae). *J. Asian Nat. Prod. Res.* **2006**, *8*, 181–185. https://doi.org/10.1080/1028602042000325564

1-(4'-hydroxy-3'-methoxyphenyl)-2-[2''-hydroxy-4''-(3-*O*-*α*-L-rhamnopyranosyloxypropyl)phenoxy]-1,3-propanediol (**27**): Popoff, T.; Theander, O. The constitutents of conifer needles. VI. Phenolic glycosides from *Pinus sylvestris*. *Acta Chem. Scand.* **1977**, *31*, 329–337. https://doi.org/10.3891/acta.chem.scand.31b-0329

(7*R*,8*S*)-3-methoxy-8,4'-oxyneoligna-3',4,7,9,9'-pentol (**28**): Gan, M.; Zhang, Y.; Lin, S.; Liu, M.; Song, W.; Zi, J.; Yang, Y.; Fan, X.; Shi, J.; Hu, J.; et al. Glycosides from the root of *Iodes cirrhosa*. *J. Nat. Prod.* **2008**, *71*, 647–654. https://doi.org/10.1021/np7007329

erythro 3-methoxy-8,4'-oxyneolignan-3',4,7,9,9'-pentol (**29**): Popoff, T.; Theander, O. The constitutents of conifer needles. VI. Phenolic glycosides from *Pinus sylvestris*. *Acta Chem. Scand.* **1977**, *31*, 329–337. https://doi.org/10.3891/acta.chem.scand.31b-0329

pinoresinol (**30**): Gabaston, J.; Richard, T.; Cluzet, S.; Palos Pinto, A.; Dufour, M.C.; Corio-Costet, M.F.; Mérillon, J.M. *Pinus pinaster* Knot: A source of polyphenols against *Plasmopara viticola*. *J. Agric. Food Chem.* **2017**, *65*, 8884–8891. https://doi.org/10.1021/acs.jafc.7b04129

(+)-salicifoliol (**31**): Chang, H.S.; Lee, S.J.; Yang, C.W.; Chen, I.S. Cytotoxic sesquiterpenes from *Magnolia kachirachirai*. *Chem. Biodivers.* **2010**, *7*, 2737–2747. https://doi.org/10.1002/cbdv.200900418

(+)-idaeusinol A (**32**): Zhou, L.; Han, F.Y.; Lu, L.W.; Yao, G.D.; Zhang, Y.Y.; Wang, X.B.; Lin, B.; Huang, X.X.; Song, S.J. Isolation of enantiomeric furolactones and furofurans from *Rubus idaeus* L. with neuroprotective activities. *Phytochemistry* **2019**, *164*, 122–129. https://doi.org/10.1016/j.phytochem.2019.05.008

schizandriside (**33**): Xu, J.F.; Li, F.S.; Feng, Z.M.; Jiang, J.S.; Zhang, P.C. A new sesquiterpenoid from *Mallotus apelta*. *Chem. Nat. Compd.* **2011**, *47*, 218–219. https://doi.org/10.1007/s10600-011-9886-4

(+)-isolariciresinol 2*α*-*O*-*α*-L-arabinoside (**34**): Popoff, T.; Theander, O. The constitutents of conifer needles. VI. Phenolic glycosides from *Pinus sylvestris*. *Acta Chem. Scand.* **1977**, *31*, 329–337. https://doi.org/10.3891/acta.chem.scand.31b-0329

(+)-isolariciresinol (**35**): Popoff, T.; Theander, O. The constitutents of conifer needles. VI. Phenolic glycosides from *Pinus sylvestris*. *Acta Chem. Scand.* **1977**, *31*, 329–337. https://doi.org/10.3891/acta.chem.scand.31b-0329

secoisolariciresinol (**36**): Gabaston, J.; Richard, T.; Cluzet, S.; Palos Pinto, A.; Dufour, M.C.; Corio-Costet, M.F.; Mérillon, J.M. *Pinus pinaster* Knot: A source of polyphenols against *Plasmopara viticola*. *J. Agric. Food Chem.* **2017**, *65*, 8884–8891. https://doi.org/10.1021/acs.jafc.7b04129

secoisolariciresinol-9,9'-acetonide (**37**): Liu, J.; Yang, C.; Zhang, J.; Wu, J.; Chen, Y. A New 5(6→7)abeo-sterol from the twigs of *Podocarpus fleuryi*. *Nat. Prod. Res.* **2017**, *31*, 175–180. https://doi.org/10.1080/14786419.2016.1224870

(–)-nortrachelogenin (**38**): Gabaston, J.; Richard, T.; Cluzet, S.; Palos Pinto, A.; Dufour, M.C.; Corio-Costet, M.F.; Mérillon, J.M. *Pinus pinaster* Knot: A source of polyphenols against *Plasmopara viticola*. *J. Agric. Food Chem.* **2017**, *65*, 8884–8891. https://doi.org/10.1021/acs.jafc.7b04129

(2*S*,3*S*)-2α-(4''-hydroxy-3''-methoxybenzyl)-3β-(4'-hydroxy-3'-methoxybenzyl)-γ-butyrolactone (**39**): Du, B.W.; Zhang, X.J.; Shi, N.; Peng, T.; Gao, J.B.; Azimova, B.; Zhang, R.; Pu, D.B.; Wang, C.; Abduvaliev, A.; et al. Luteolin-7-methylether from *Leonurus japonicus* inhibits estrogen biosynthesis in human ovarian granulosa cells by suppression of aromatase (CYP19). *Eur. J. Pharmacol.* **2020**, *879*, 173154. https://doi.org/10.1016/j.ejphar.2020.173154

astragalin (**40**): Luyen, B.T.; Tai, B.H.; Thao, N.P.; Eun, K.J.; Cha, J.Y.; Xin, M.J.; Lee, Y.M.; Kim, Y.H. Anti-inflammatory components of *Euphorbia humifusa* Willd. *Bioorg. Med. Chem. Lett.* **2014**, *24*, 1895–1900. https://doi.org/10.1016/j.bmcl.2014.03.014

kaempferol-3-*O*-β-D-galactopyranoside (**41**): Scharbert, S.; Holzmann, N.; Hofmann, T. Identification of the astringent taste compounds in black tea infusions by combining instrumental analysis and human bioresponse. *J. Agric. Food Chem.* **2004**, *52*, 3498–3508. https://doi.org/10.1021/jf049802u

kaempferol-3-*O*-α-L-furanoarabinoside (**42**): Wang, P.H.; Lee, S.S. Polar chemical constituents from *Phoebe formosana*. *J. Chin. Chem. Soc.* **1999**, *46*, 215–219. https://doi.org/10.1002/jccs.199900034

rhamnetin 3-*O*-β-D-glucopyranoside (**43**): He, Z.; Lian, W.; Liu, J.; Zheng, R.; Xu, H.; Du, G.; Liu, A. Isolation, structural characterization and neuraminidase inhibitory activities of polyphenolic constituents from *Flos caryophylli*. *Phytochem. Lett.* **2017**, *19*, 160–167. https://doi.org/10.1016/j.phytol.2016.12.031

apigenin (**44**): Xu, Y.; Tao, Z.; Jin, Y.; Yuan, Y.; Dong, T.T.X.; Tsim, K.W.K.; Zhou, Z. Flavonoids, a potential new insight of *Leucaena leucocephala* foliage in ruminant health. *J. Agric. Food Chem.* **2018**, *66*, 7616–7626. https://doi.org/10.1021/acs.jafc.8b02739

kaempferol-3,6-dimethyl ether (**45**): Rabesa, Z.A.; Voirin, B. Un nouvel aglycone *C*-methyl flavonique, le *C*-methyl-6 0-methyl-3 kaempferol isole de alluaudia dumosa. *Tetrahedron Lett.* **1978**, *19*, 3717–3718. https://doi.org/10.1016/S0040-4039(01)95039-7

5,7,8,4'-tetrahydroxy-3-methoxy-6-methylflavonol-8-*O*-*β*-D-glucopyranoside (**46**): Jung, M.J.; Choi, J.H.; Chung, H.Y.; Jung, J.H.; Choi, J.S. A new *C*-methylated flavonoid glycoside from *Pinus densiflora*. *Fitoterapia* **2001**, *72*, 943–945. https://doi.org/10.1016/S0367-326X(01)00337-9

6-methylaromadendrin (**47**): Shen, Z.; Theander, O. Flavonoid glycosides from needles of *Pinus massoniana*. *Phytochemistry* **1985**, *24*, 155–158. https://doi.org/10.1016/S0031-9422(00)80826-2

naringenin (**48**): Xu, Y.; Tao, Z.; Jin, Y.; Yuan, Y.; Dong, T.T.X.; Tsim, K.W.K.; Zhou, Z. Flavonoids, a potential new insight of *Leucaena leucocephala* foliage in ruminant health. *J. Agric. Food Chem.* **2018**, *66*, 7616–7626. https://doi.org/10.1021/acs.jafc.8b02739

tiliroside (**49**): Hendra, R.; Keller, P.A. Phytochemical studies on two Australian *Anigozanthos* plant species. *J. Nat. Prod.* **2017**, *80*, 2141–2145. https://doi.org/10.1021/acs.jnatprod.7b00063

kaempferol 3-*O*-(3'',6''-di-*O*-*E*-*p*-coumaroyl)-*β*-D-glucopyranoside (**50**): Sa, N.H.; Tam, N.T.; Anh, N.T.H.; Quan, T.D.; Thien, D.D.; Phong, D.T.; Sung, T.V.; Thuy, T.T. Chemical constituents from the leaves of *Pinus dalatensis* Ferré. *Nat. Prod. Res.* **2018**, *32*, 341–345. https://doi.org/10.1080/14786419.2017.1350672

kaempferol-3-*O*-(5''-*O*-*E*-*p*-coumaroyl)-*α*-L-arabinofuranoside (**51**): Gao, Y.; Yuan, J.Z.; Wang, Y.X.; Zhang, B.K.; Sun, Q.S. Isolation and identification of flavonoids from pine needle of *Pinus koraiensis* Sieb. et Zucc. *Shenyang yao ke da xue xue bao* **2010**, *27*, 539–543.

machilusoxide A (**52**): Tai, S.H.; Kuo, P.C.; Lam, S.H.; Huang, S.C.; Kuo, Y.Z.; Hung, H.Y.; Liou, M.J.; Shieh, P.C.; Lee, E.J.; Wu, T.S. Chemical constituents from the stems of *Machilus philippinensis* Merr. and the neuroprotective activity of cinnamophilin. *RSC Adv.* **2019**, *9*, 21616–21625. https://doi.org/10.1039/C9RA03514A

(+)-(*S*)-dehydrovomifoliol (**53**): Xiao, Y.; Zhu, S.; Wu, G.; ul Hassan, S.S.; Xie, Y.; Ishaq, M.; Sun, Y.; Yan, S.K.; Qian, X.P.; Jin, H.Z. Chemical constituents of *Vernonia parishii*. *Chem. Nat. Compd.* **2020**, *56*, 134–136. https://doi.org/10.1007/s10600-020-02963-x

isololiolide (**54**): Kimura, J.; Maki, N. New loliolide derivatives from the brown alga *Undaria pinnatifida*. *J. Nat. Prod.* **2002**, *65*, 57–58. https://doi.org/10.1021/np0103057

(*S*)-(+)-abscisic acid sodium salt (**55**): Abrams, S.R.; Reaney, M.J.T.; Abrams, G.D.; Mazurek, T.; Shaw, A.C.; Gusta, L.V. Ratio of (*S*)- to (*R*)-abscisic acid from plant cell cultures supplied with racemic aba. *Phytochemistry* **1989**, *28*, 2885–2889. https://doi.org/10.1016/0031-9422(89)80246-8

(3*S*,5*R*,6*R*,7*E*)-3,5,6-trihydroxy-7-megastigmen-9-one (**56**): Cai, X.; Liu, J.X.; Song, Q.S.; Chen, K.L.; Lu, Z.Y.; Zhang, Y.M. Chemical constituents of *Afzelia xylocarpa*. *Chem. Nat. Compd.* **2018**, *54*, 764–765. https://doi.org/10.1007/s10600-018-2467-z

blumenol A (**57**): Afolayan, M.; Srivedavyasasri, R.; Asekun, O.T.; Familoni, O.B.; Ross, S.A. Chemical

and biological studies on *Bridelia ferruginea* grown in Nigeria. *Nat. Prod. Res.* **2019**, *33*, 287–291. https://doi.org/10.1080/14786419.2018.1440225

peltopterin B (**58**): Li, Y.C.; Kuo, P.C.; Yang, M.L.; Chen, T.Y.; Hwang, T.L.; Chiang, C.C.; Thang, T.D.; Tuan, N.N.; Tzen, J.T.C. Chemical constituents of the leaves of *Peltophorum pterocarpum* and their bioactivity. *Molecules* **2019**, *24*, 240. https://dx.doi.org/10.3390%2Fmolecules24020240

3,4-dihydroxybenzoic acid methyl ester (**59**): Kim, T.H.; Lee, J.; Kim, H.J.; Jo, C. Plasma-induced degradation of quercetin associated with the enhancement of biological activities. *J. Agric. Food Chem.* **2017**, *65*, 6929–6935. https://doi.org/10.1021/acs.jafc.7b00987

p-hydroxybenzoic acid (**60**): Xin, X.L.; Aisa, H.A.; Wang, H.Q. Flavonoids and phenolic compounds from seeds of the Chinese plant *Nigella glandulifera*. *Chem. Nat. Compd.* **2008**, *44*, 368–369. https://doi.org/10.1007/s10600-008-9066-3

vanillic acid (**61**): Nishanbaev, S.Z.; Bobakulov, K.M.; Abdullaev, N.D.; Sham'yanov, I.D. Phenolcarboxylic acids from *Quercus robur* growing in Uzbekistan. *Chem. Nat. Compd.* **2015**, *51*, 537–539. https://doi.org/10.1007/s10600-015-1334-4

4-hydroxybenzaldehyde (**62**): Chung, C.P.; Hsia, S.M.; Lee, M.Y.; Chen, H.J.; Cheng, F.; Chan, L.C.; Kuo, Y.H.; Lin, Y.L.; Chiang, W. Gastroprotective activities of adlay (*Coix lachryma-jobi* L. var. *ma-yuen* Stapf) on the growth of the stomach cancer AGS cell line and indomethacin-induced gastric ulcers. *J. Agric. Food Chem.* **2011**, *59*, 6025–6033. https://doi.org/10.1021/jf2009556

methylparaben (**63**): Xin, X.L.; Aisa, H.A.; Wang, H.Q. Flavonoids and phenolic compounds from seeds of the Chinese plant *Nigella glandulifera*. *Chem. Nat. Compd.* **2008**, *44*, 368–369. https://doi.org/10.1007/s10600-008-9066-3

3-hydroxy-1-(4-hydroxy-3-methoxyphenyl)-1-propanone (**64**): Ni, J.C.; Shi, J.T.; Tan, Q.W.; Chen, Q.J. Two new compounds from the fruit of *Ailanthus altissima*. *Nat. Prod. Res.* **2019**, *33*, 101–107. https://doi.org/10.1080/14786419.2018.1437434

3-hydroxy-1-(4-hydroxyphenyl)-1-propanone (**65**): Ni, J.C.; Shi, J.T.; Tan, Q.W.; Chen, Q.J. Two new compounds from the fruit of *Ailanthus altissima*. *Nat. Prod. Res.* **2019**, *33*, 101–107. https://doi.org/10.1080/14786419.2018.1437434

2-(4-hydroxyphenyl)acetic acid (**66**): Gil Archila, E.; Cuca Suárez, L.E. Phytochemical study of leaves of *Ocotea caudata* from Colombia. *Nat. Prod. Res.* **2018**, *32*, 195–201. https://doi.org/10.1080/14786419.2017.1344663

phenylacetic acid (**67**): Gachet, M.S.; Kunert, O.; Kaiser, M.; Brun, R.; Muñoz, R.A.; Bauer, R.; Schühly, W. Jacaranone-derived glucosidic esters from *Jacaranda glabra* and their activity against *Plasmodium falciparum*. *J. Nat. Prod.* **2010**, *73*, 553–556. https://doi.org/10.1021/np900528m

isovanillic acid (**68**): Ding, H.Y.; Lin, H.C.; Teng, C.M.; Wu, Y.C. Phytochemical and pharmacological studies on Chinese *Paeonia* Species. *J. Chin. Chem. Soc.* **2000**, *47*, 381–388. https://doi.org/10.1002/jccs.200000051

benzoic acid (**69**): Laurent, P.; Lebrun, B.; Braekman, J.-C.; Daloze, D.; Pasteels, J.M. Biosynthetic studies on adaline and adalinine, two alkaloids from ladybird beetles (Coleoptera: Coccinellidae). *Tetrahedron* **2001**, *57*, 3403–3412. https://doi.org/10.1016/S0040-4020(01)00207-1

vanillin (**70**): Chung, C.P.; Hsia, S.M.; Lee, M.Y.; Chen, H.J.; Cheng, F.; Chan, L.C.; Kuo, Y.H.; Lin, Y.L.; Chiang, W. Gastroprotective activities of adlay (*Coix lachryma-jobi* L. var. *ma-yuen* Stapf) on the growth of the stomach cancer AGS cell line and indomethacin-induced gastric ulcers. *J. Agric. Food Chem.* **2011**, *59*, 6025–6033. https://doi.org/10.1021/jf2009556

p-hydroxyacetophenone (**71**): Xiu, F.; Li, X.; Zhang, W.; He, F.; Ying, X.; Stien, D. A new alkaloid from *Portulaca oleracea* L. and its antiacetylcholinesterase activity. *Nat. Prod. Res.* **2019**, *33*, 2583–2590. https://doi.org/10.1080/14786419.2018.1460833

sodium salicylate (**72**): Manohar, C.; Rao, U.R.K.; Valaulikar, B.S.; Lyer, R.M. On the origin of viscoelasticity in micellar solutions of cetyltrimethylammonium bromide and sodium salicylate. *J. Chem. Soc. Chem. Commun.* **1986**, 379–381. https://doi.org/10.1039/C39860000379

vannilic acid 4-*O*-*α*-L-rhamnoside (**73**): Termentzi, A.; Zervou, M.; Kokkalou, E. Isolation and structure elucidation of novel phenolic constituents from *Sorbus domestica* fruits. *Food Chem.* **2009**, *116*, 371–381. https://doi.org/10.1016/j.foodchem.2009.02.019

trans-ferulic acid (**74**): Prachayasittikul, S.; Suphapong, S.; Worachartcheewan, A.; Lawung, R.; Ruchirawat, S.; Prachayasittikul, V. Bioactive metabolites from *Spilanthes acmella* Murr. *Molecules* **2009**, *14*, 850–867. https://doi.org/10.3390/molecules14020850

sodium *p*-coumarate (**75**): Lam, S.H.; Li, Y.C.; Kuo, P.C.; Hwang, T.L.; Yang, M.L.; Wang, C.C.; Tzen, J.T.C. Chemical constituents of *Vigna luteola* and their anti-inflammatory bioactivity. *Molecules* **2019**, *24*, 1371. https://dx.doi.org/10.3390%2Fmolecules24071371

p-coumaric acid (**76**): Costa, A.G.; Yoshida, N.C.; Garcez, W.S.; Perdomo, R.T.; Matos, M.d.F.C.; Garcez, F.R. Metabolomics approach expands the classification of propolis samples from midwest Brazil. *J. Nat. Prod.* **2020**, *83*, 333–343. https://doi.org/10.1021/acs.jnatprod.9b00783

trans-methyl *p*-coumarate (**77**): Gopalakrishnan, S.; Subbarao, G.V.; Nakahara, K.; Yoshihashi, T.; Ito, O.; Maeda, I.; Ono, H.; Yoshida, M. Nitrification inhibitors from the root tissues of *Brachiaria humidicola*, a tropical grass. *J. Agric. Food Chem.* **2007**, *55*, 1385–1388. https://doi.org/10.1021/jf062593o

Table S1. Preliminary bioactivity screening of needles of *P. taiwanensis* on superoxide anion generation and elastase release by human neutrophils in response to fMLF/CB.

Samples	Superoxide Anion Generation		Elastase Release	
	IC_{50} (μg/mL) [a]	Inh % [b]	IC_{50} (μg/mL)	Inh %
Methanol extract	– [c]	14.6 ± 6.3	–	11.1 ± 5.7
n-hexane layer	–	27.2 ± 5.8 **	–	17.4 ± 6.4 *
Ethyl acetate layer	0.8 ± 0.2	103.0 ± 0.5 ***	1.0 ± 0.1	121.2 ± 1.9 ***
Water layer	–	26.2 ± 3.4 ***	–	23.8 ± 9.2 *
LY294002 [d]	1.1 ± 0.3	100.6 ± 1.0 ***	3.2 ± 1.0	76.7 ± 6.8 ***

Results are presented as mean ± SEM (n = 3-5). * $p < 0.05$, ** $p < 0.01$, *** $p < 0.001$ compared with the control (DMSO). [a] Concentration necessary for 50 % inhibition (IC_{50}). [b] Percentage of inhibition (Inh %) at 10 μg/mL concentration. [c] Not determined. [d] A phosphatidylinositol-3-kinase inhibitor was used as a positive control.

Table S2. Inhibitory effects of purified compounds on superoxide anion generation and elastase release by human neutrophils in response to fMLF/CB.

compound	Superoxide anion generation		Elastase release	
	IC_{50} (μM) [a]	Inh % [b]	IC_{50} (μM)	Inh %
**1**	– [c]	19.6 ± 5.5 *	–	15.9 ± 4.1 *
**2**	–	11.7 ± 3.3 *	–	10.3 ± 4.5
**3**	–	24.7 ± 4.7 **	–	23.5 ± 6.2 *
**4**	–	22.9 ± 5.6 **	–	10.2 ± 3.8
**5**	–	24.0 ± 3.8 **	–	11.1 ± 4.5
**10**	–	4.7 ± 1.7 *	–	5.4 ± 2.3
**11**	–	4.9 ± 2.4	–	8.4 ± 3.4
**12**	–	9.7 ± 4.0	–	4.8 ± 1.2 *
**13**	–	3.7 ± 2.4	–	3.6 ± 4.1
**14**	–	15.6 ± 6.3 *	–	6.6 ± 6.7
**15**	–	17.1 ± 5.0 *	–	5.8 ± 5.6
**16**	–	5.7 ± 0.8 **	–	1.7 ± 3.5
**17**	–	2.8 ± 2.9	–	9.4 ± 7.0
**18**	–	43.2 ± 7.1 **	–	29.8 ± 6.1 **
**19**	–	28.9 ± 6.9 **	–	4.0 ± 1.8
**20**	–	16.6 ± 3.7 *	–	11.0 ± 4.4
**22**	–	19.4 ± 3.0 **	–	14.1 ± 4.9 *
**23**	–	13.2 ± 5.9	–	5.0 ± 2.3
**24**	–	10.3 ± 4.4	–	20.7 ± 6.3 *
**25**	–	7.3 ± 2.1 *	–	13.4 ± 6.5
**26**	–	23.9 ± 5.9 *	–	-1.2 ± 3.5
**27**	–	13.2 ± 4.6 *	–	-2.3 ± 2.3
**28**	–	7.5 ± 3.2	–	10.4 ± 6.3
**29**	–	15.0 ± 4.4 *	–	4.5 ± 5.0
**31**	–	34.4 ± 6.1 **	–	3.5 ± 3.5
**32**	–	12.5 ± 6.1	–	9.4 ± 3.7
**33**	–	20.0 ± 5.8 *	–	13.3 ± 2.5 **
**34**	–	16.3 ± 3.4 **	–	3.3 ± 2.9
**35**	–	27.1 ± 6.4 **	–	6.3 ± 2.8
**36**	–	23.0 ± 6.9 *	–	12.4 ± 4.2 *
**37**	–	49.7 ± 6.4 ***	–	8.4 ± 6.8
**38**	–	34.2 ± 4.8 ***	–	14.0 ± 6.4
**39**	–	45.5 ± 7.0 ***	–	30.8 ± 5.2 **
**40**	–	9.3 ± 4.8	–	3.9 ± 4.1
**41**	–	17.0 ± 7.0	–	2.7 ± 3.0
**42**	–	28.3 ± 4.7 **	–	6.2 ± 6.3
**45**	6.4 ± 0.7	70.5 ± 6.8 ***	–	34.2 ± 6.9 **
**46**	–	13.7 ± 5.2 *	–	38.6 ± 6.2 **
**47**	6.0 ± 1.1	71.8 ± 8.1 ***	–	43.5 ± 6.9 ***
**48**	3.3 ± 0.9	87.5 ± 5.4 ***	5.3 ± 0.2	93.9 ± 5.2 ***
**49**	7.7 ± 0.9	60.6 ± 3.9 ***	–	40.3 ± 6.0 **
**50**	5.3 ± 1.1	72.9 ± 6.3 ***	5.8 ± 0.9	81.4 ± 12.0 ***
**51**	–	45.2 ± 5.6 ***	8.3 ± 0.8	57.0 ± 4.6 ***
**LY294002** [d]	1.1 ± 0.3	100.6 ± 1.0 ***	3.2 ± 1.0	76.7 ± 6.8 ***

Results are presented as mean ± SEM (n = 3–5). $*p < 0.05$, $**p < 0.01$, $***p < 0.001$ compared with the control (DMSO). [a] Concentration necessary for 50 % inhibition (IC_{50}). [b] Percentage of inhibition (Inh %) at 10 μM concentration. [c] Not determined. [d] A phosphatidylinositol-3-kinase inhibitor was used as a positive control.

Figure S1. HR-ESI-MS pectrum of **1**.

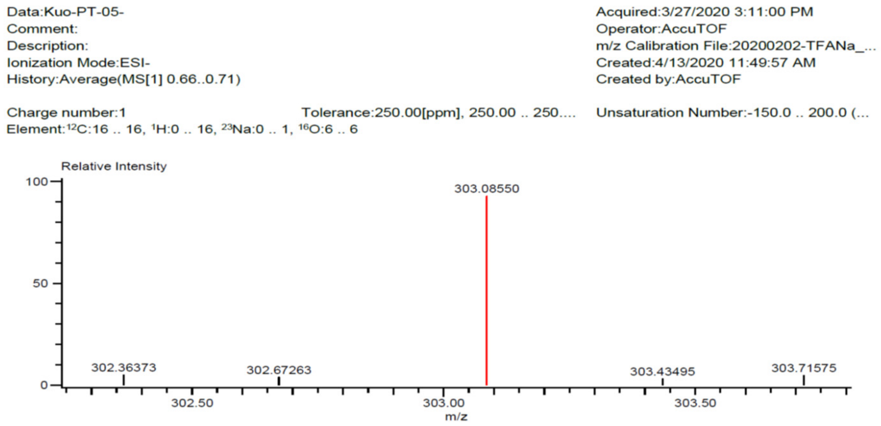

Mass	Intensity	Calc. Mass	Mass Difference [mDa]	Mass Difference [ppm]	Possible Formula
303.08550	1979.22	303.08686	-1.37	-4.51	$^{12}C_{16}{}^{1}H_{15}{}^{16}O_{6}$

Figure S2. ^{1}H NMR spectrum of **1** (CD_3OD, 400 MHz).

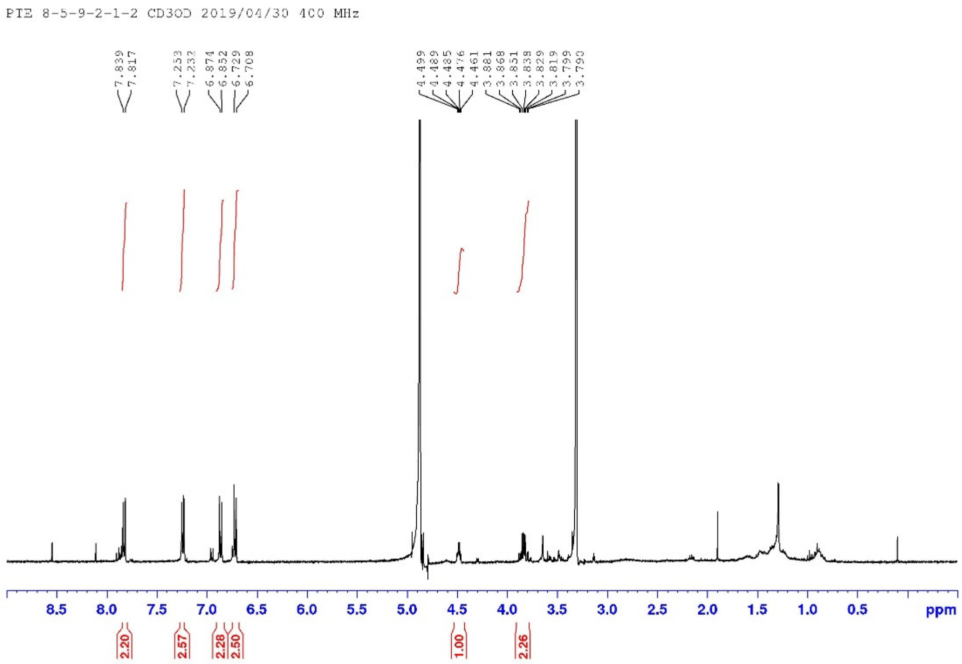

Figure S3. ^{13}C and DEPT spectrum of **1** (CD_3OD, 100 MHz).

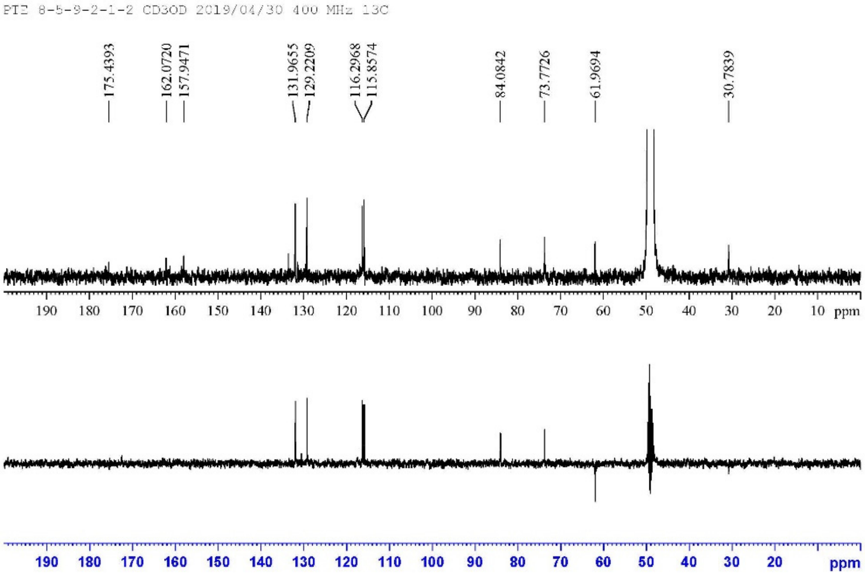

Figure S4. HMBC spectrum of **1** (CD_3OD, 400 MHz).

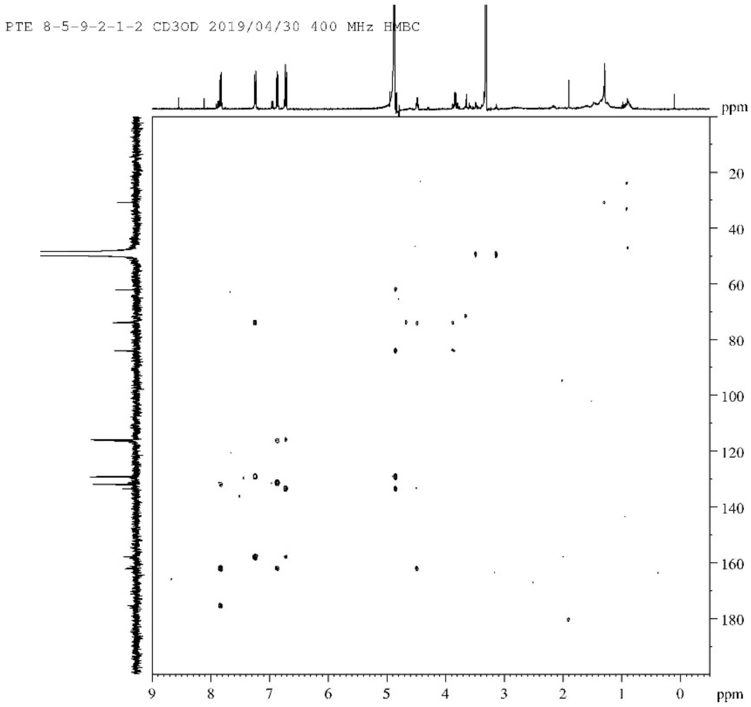

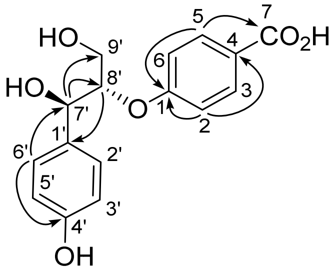

Figure S5. NOESY spectrum of **1** (CD_3OD, 400 MHz).

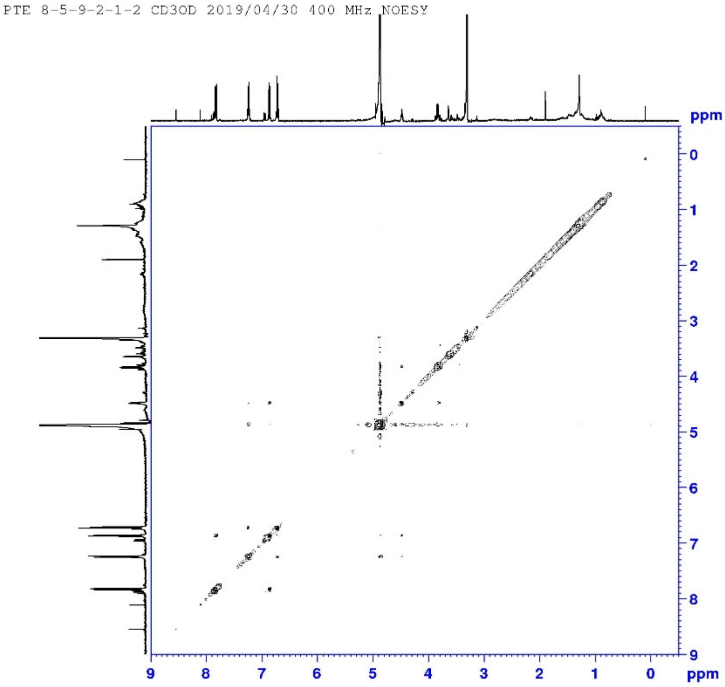

Figure S6. COSY spectrum of **1** (CD_3OD, 400 MHz).

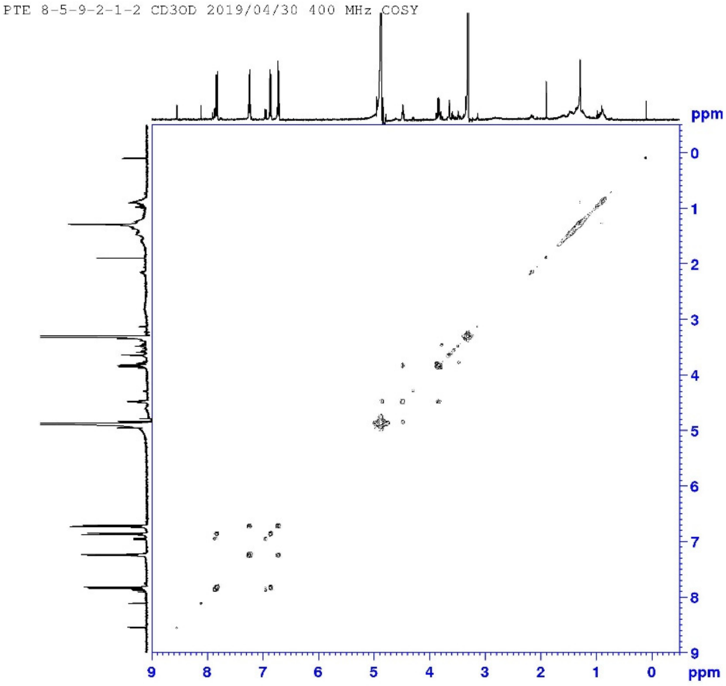

Figure S7. HSQC spectrum of **1** (CD_3OD, 400 MHz).

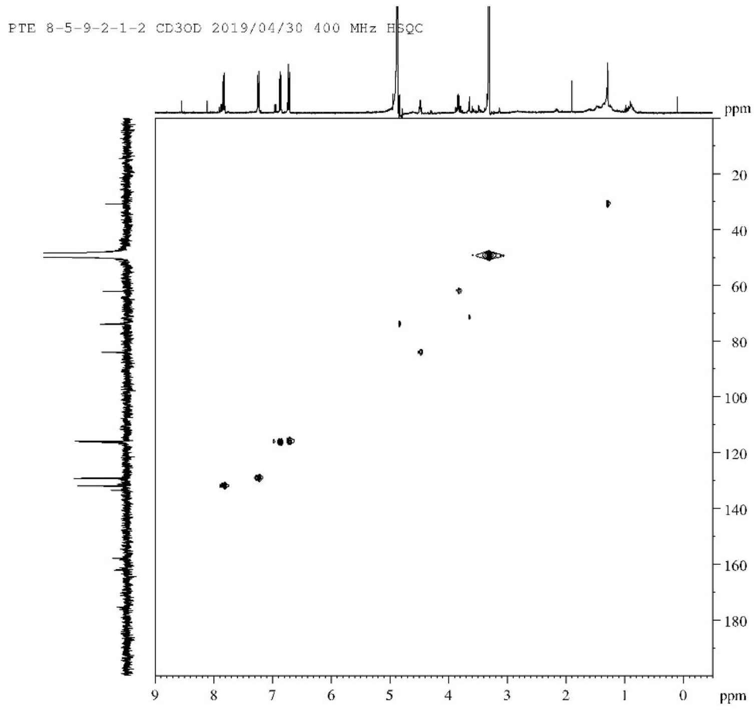

Figure S8. HR-ESI-MS spectrum of **2**.

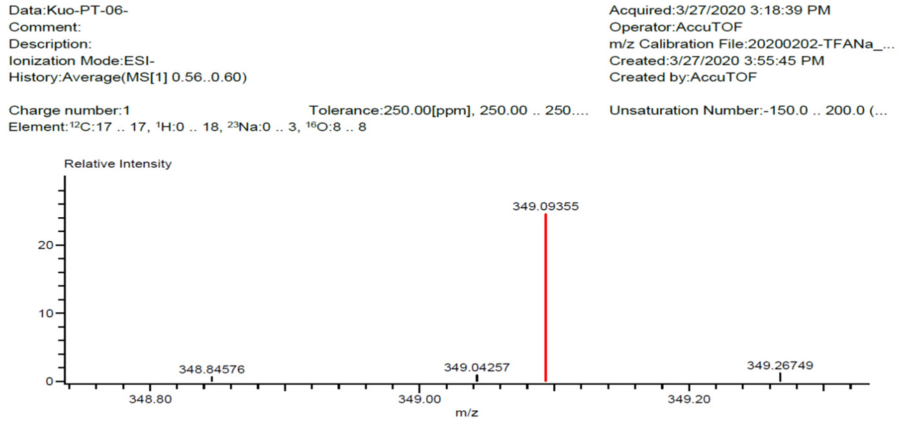

Mass	Intensity	Calc. Mass	Mass Difference [mDa]	Mass Difference [ppm]	Possible Formula
349.09355	1937.42	349.09234	1.21	3.47	$^{12}C_{17}{}^{1}H_{17}{}^{16}O_8$

Figure S9. ^{1}H NMR spectrum of **2** (CD_3OD, 400 MHz).

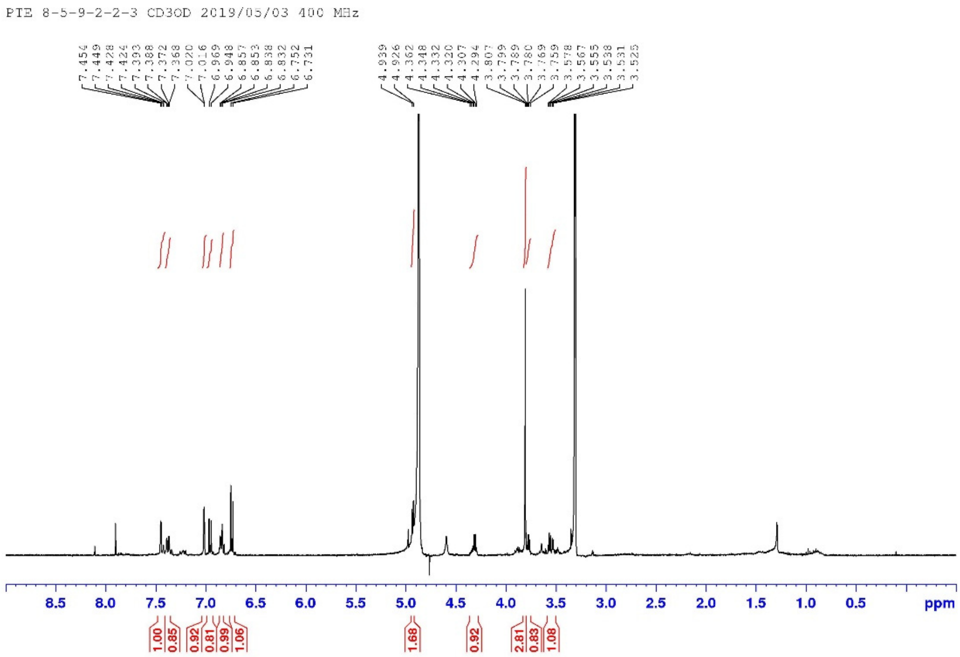

Figure S10. ^{13}C and DEPT NMR spectrum of **2** (CD_3OD, 100 MHz).

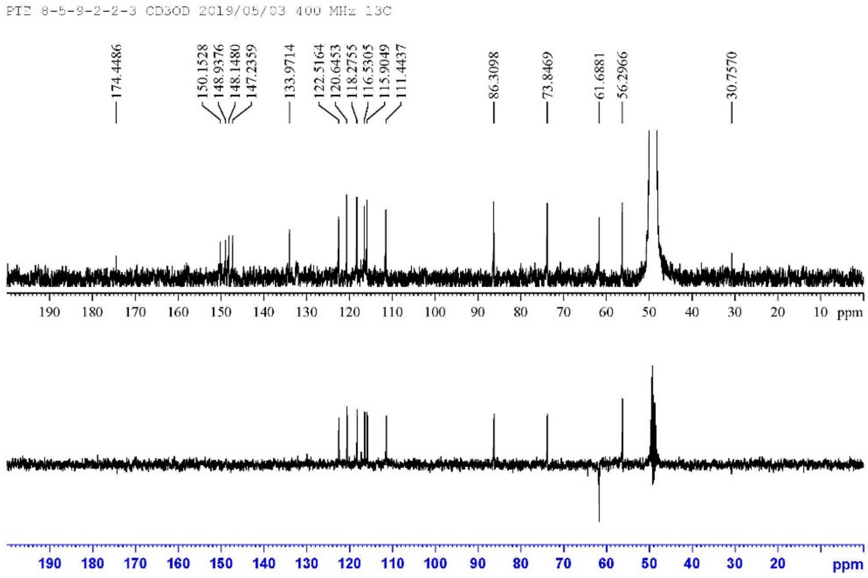

Figure S11. HMBC spectrum of **2** (CD_3OD, 400 MHz).

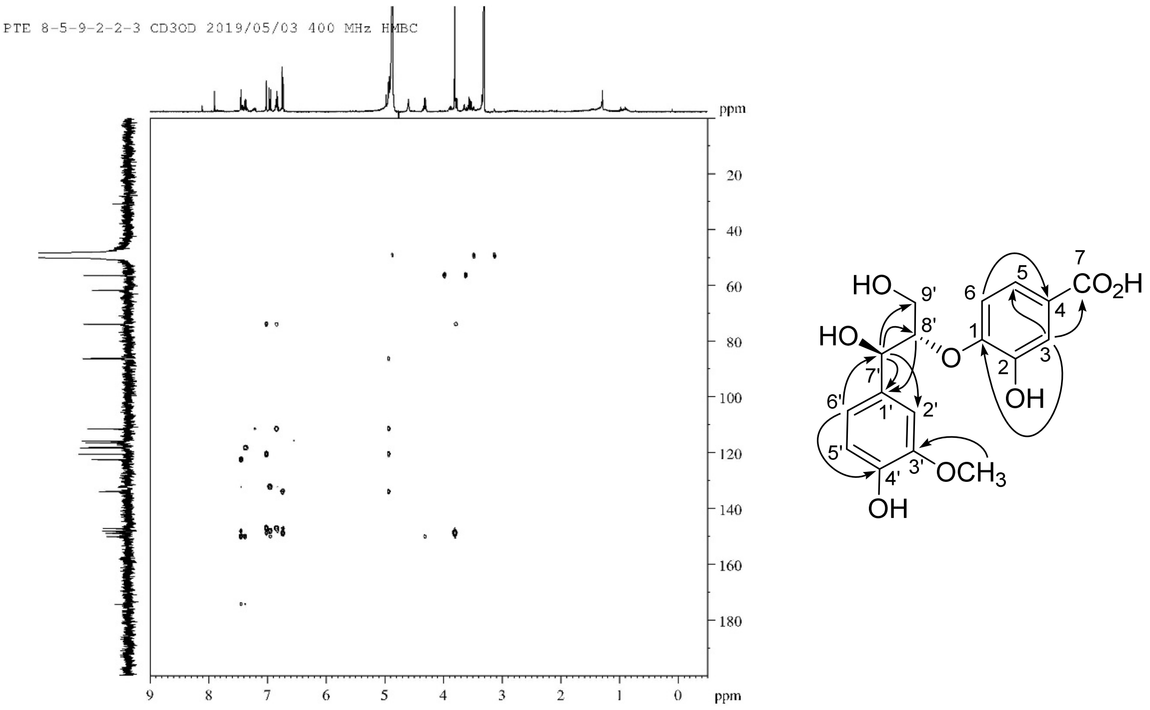

Figure S12. NOESY spectrum of **2** (CD_3OD, 400 MHz).

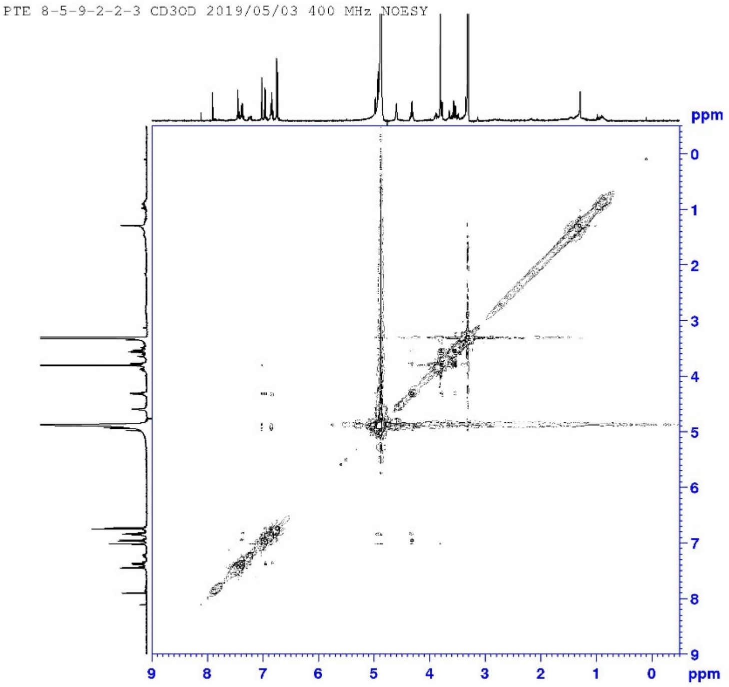

Figure S13. COSY spectrum of **2** (CD_3OD, 400 MHz).

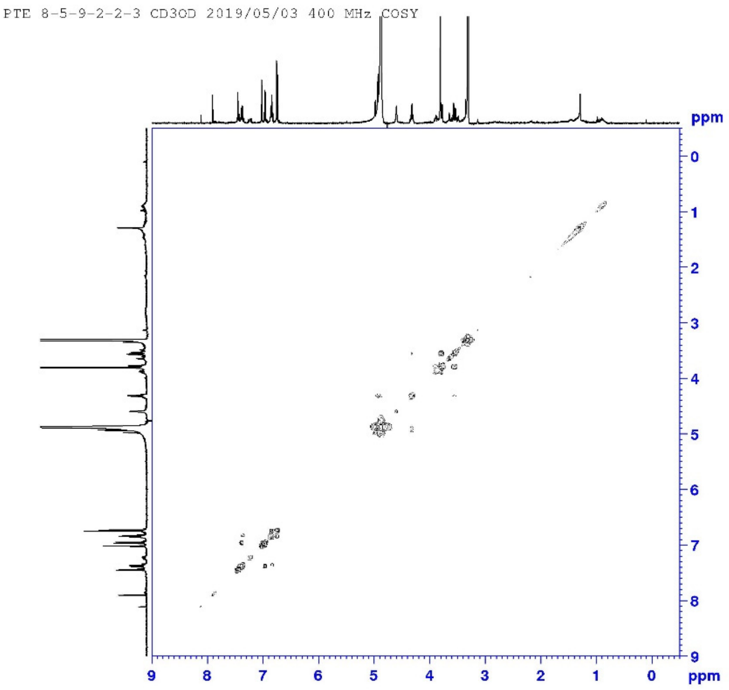

Figure S14. HSQC spectrum of **2** (CD_3OD, 400 MHz).

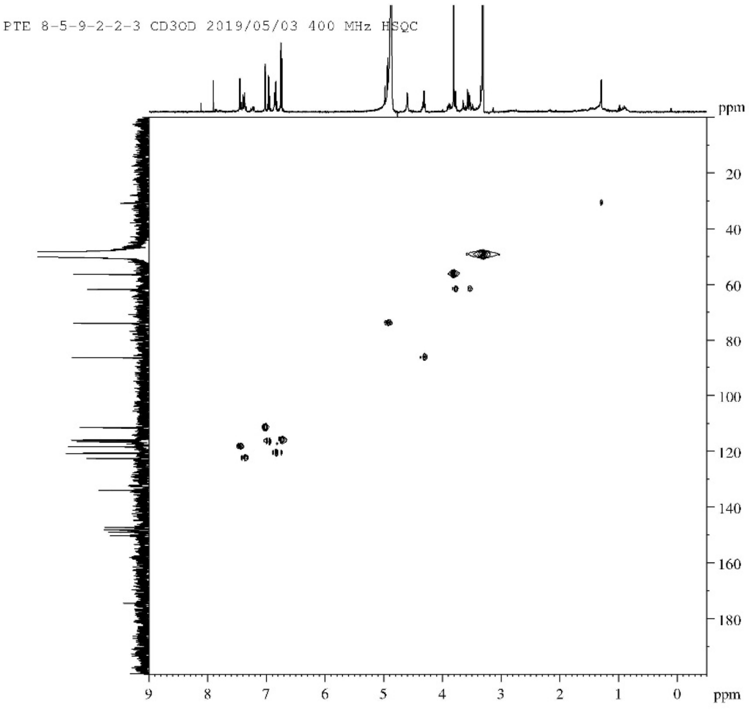

Figure S15. HR-ESI-MS spectrum of **3**.

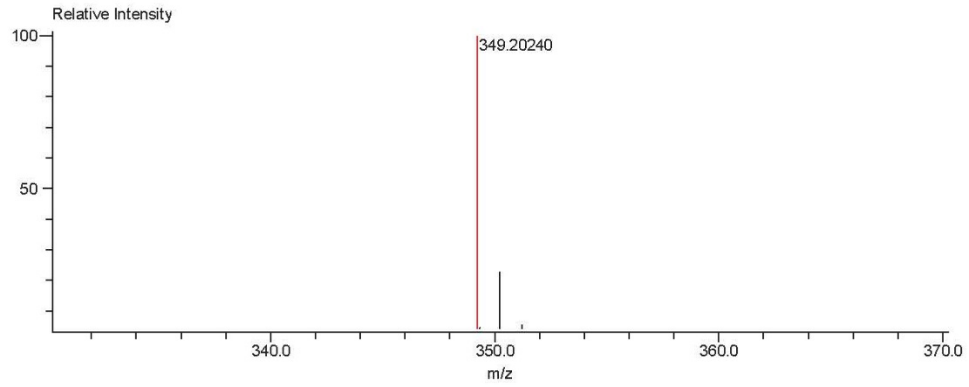

Mass	Intensity	Calc. Mass	Mass Difference [mDa]	Mass Difference [ppm]	Possible Formula
349.20240	15073.70	349.20150	0.90	2.59	$^{12}C_{20}{}^{1}H_{29}{}^{16}O_5$

Figure S16. ^{13}C and DEPT NMR spectrum of **3** (CD_3OD, 100 MHz).

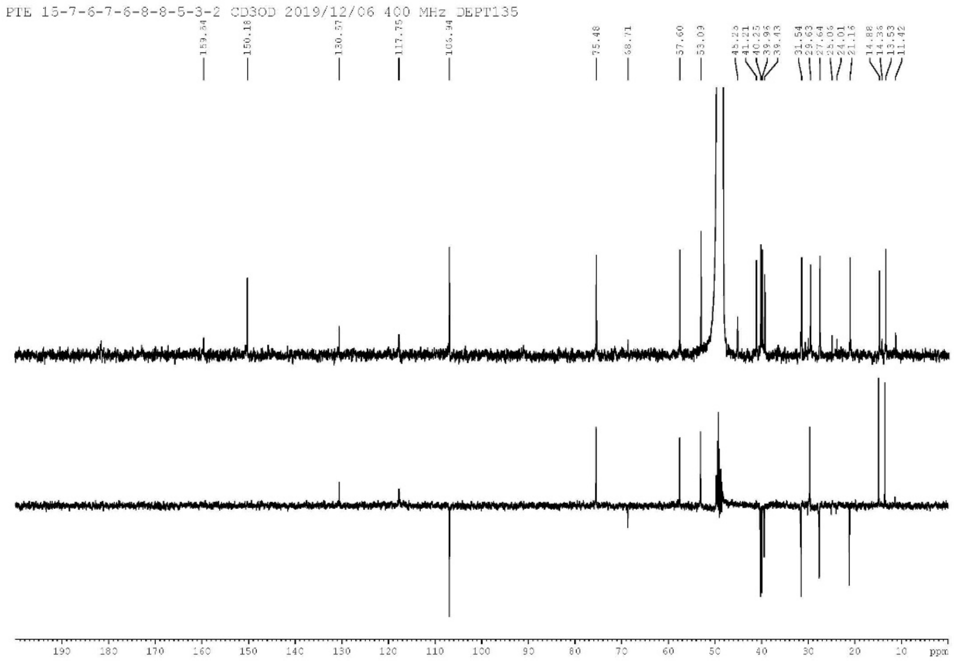

Figure S17. ^{1}H NMR spectrum of **3** (CD_3OD, 400 MHz).

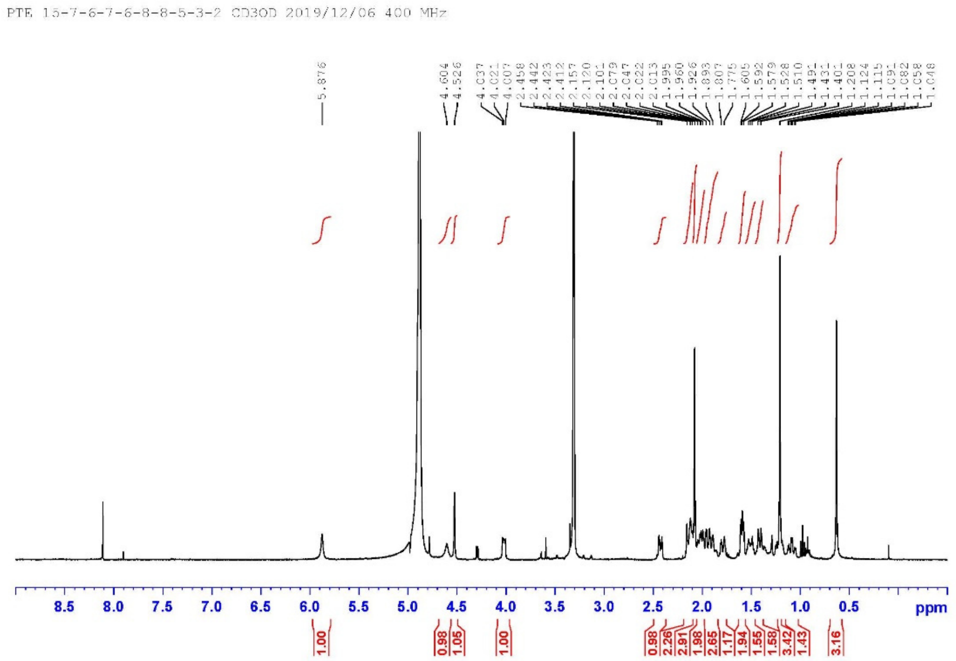

Figure S18. HMBC spectrum of **3** (CD_3OD, 400 MHz).

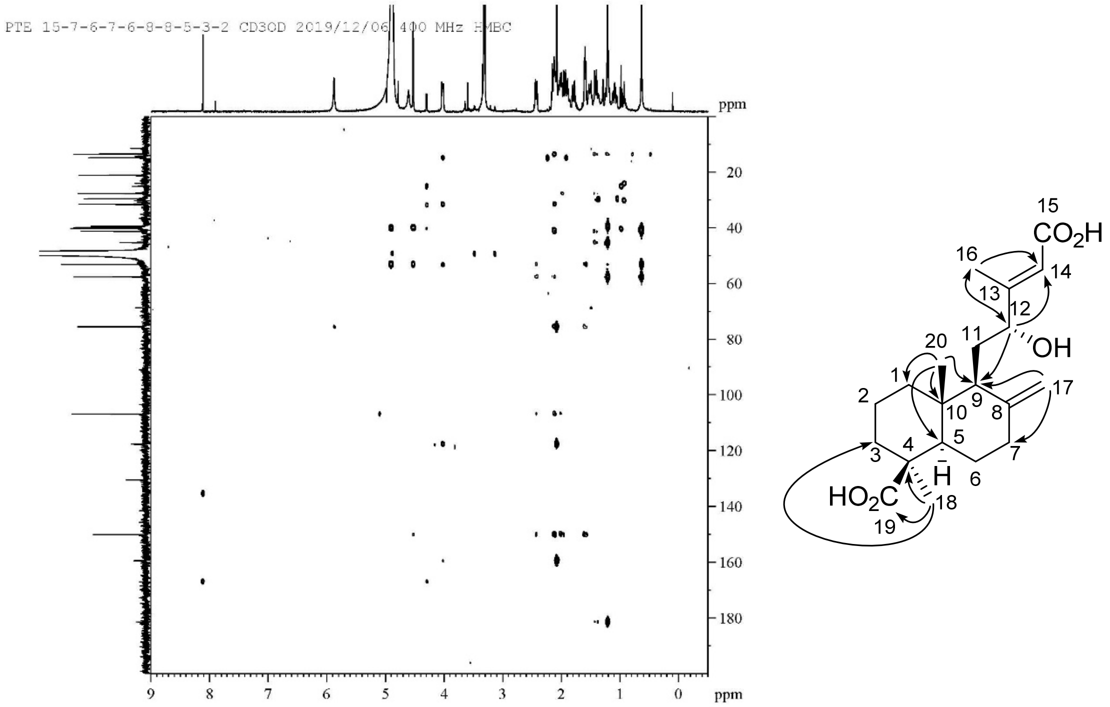

Figure S19. NOESY spectrum of **3** (CD_3OD, 400 MHz).

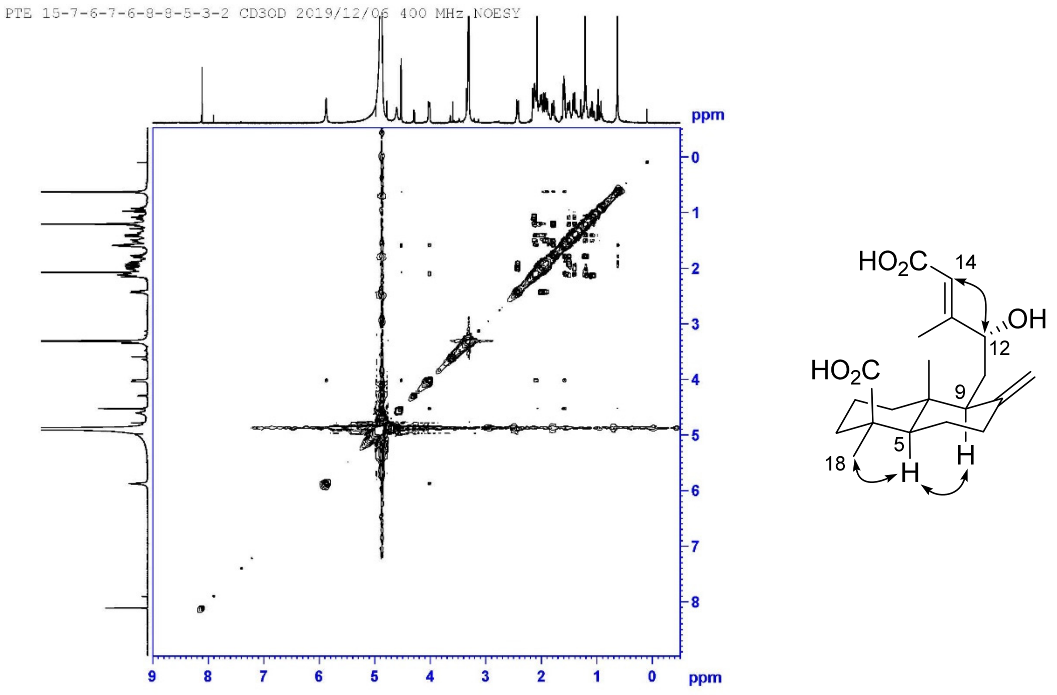

Figure S20. COSY spectrum of **3** (CD_3OD, 400 MHz).

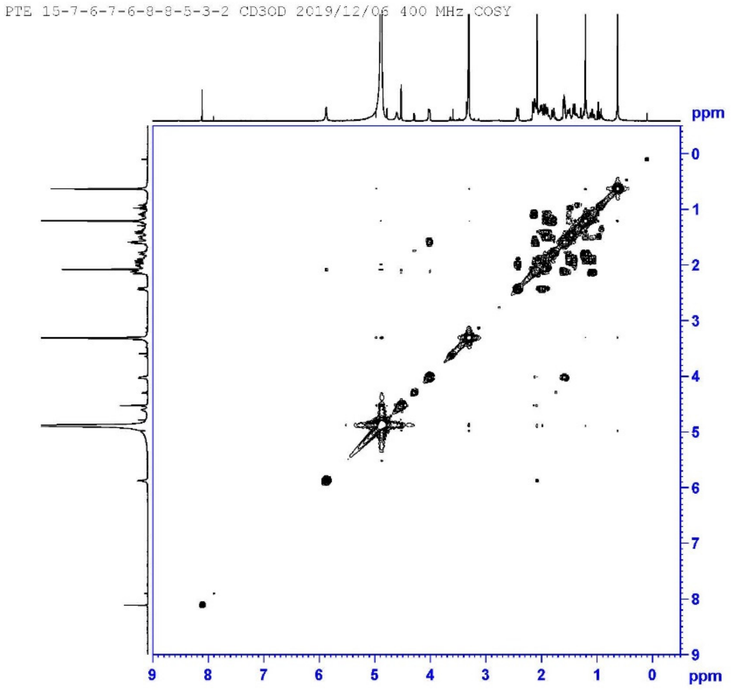

Figure S21. HSQC spectrum of **3** (CD_3OD, 400 MHz).

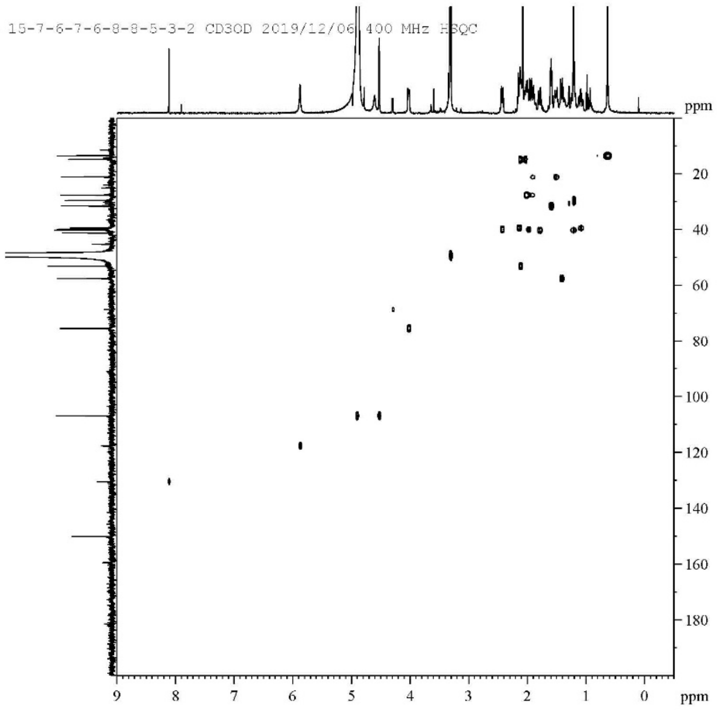

Figure S22. HR-ESI-MS spectrum of **4**.

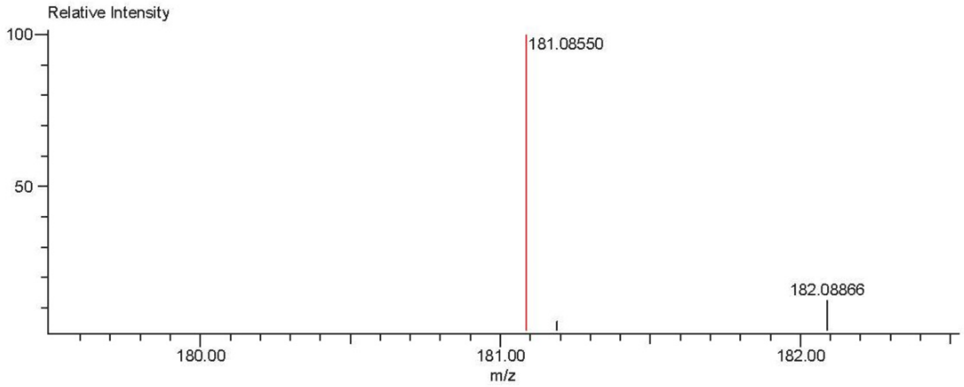

Mass	Intensity	Calc. Mass	Mass Difference [mDa]	Mass Difference [ppm]	Possible Formula
181.08550	35646.11	181.08647	-0.97	-5.36	$^{12}C_{10}{}^{1}H_{13}{}^{16}O_3$

Figure S23. ^{1}H NMR spectrum of **4** (CD_3OD, 400 MHz).

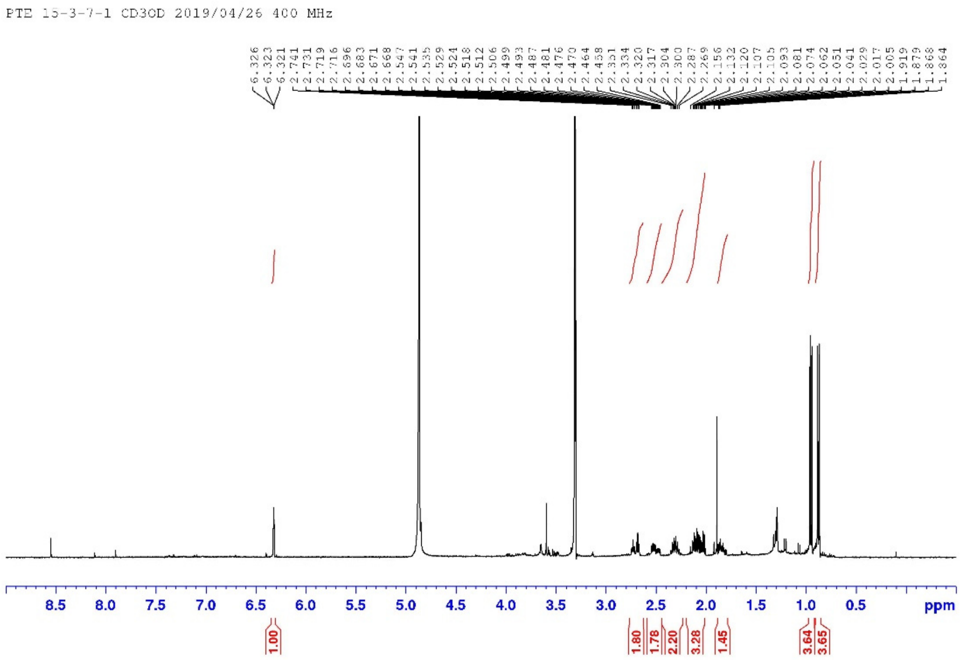

Figure S24. ^{13}C and DEPT NMR spectrum of **4** (CD_3OD, 100 MHz).

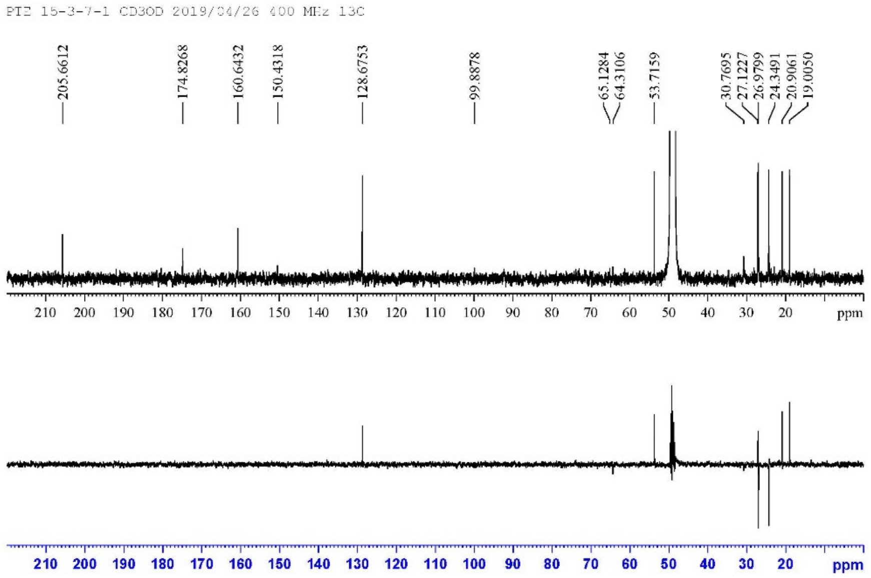

Figure S25. HMBC spectrum of **4** (CD_3OD, 400 MHz).

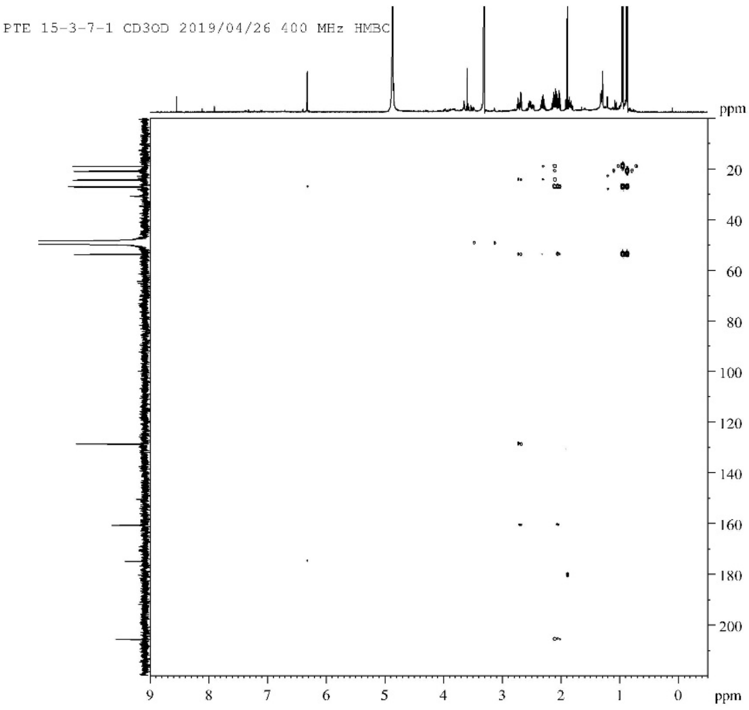

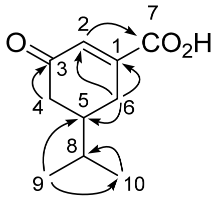

Figure S26. NOESY spectrum of **4** (CD_3OD, 400 MHz).

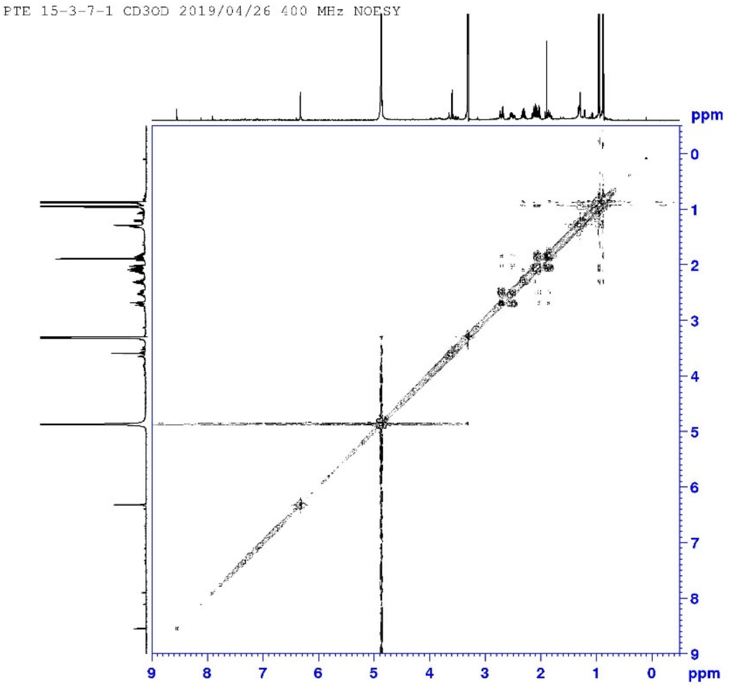

Figure S27. COSY spectrum of **4** (CD_3OD, 400 MHz).

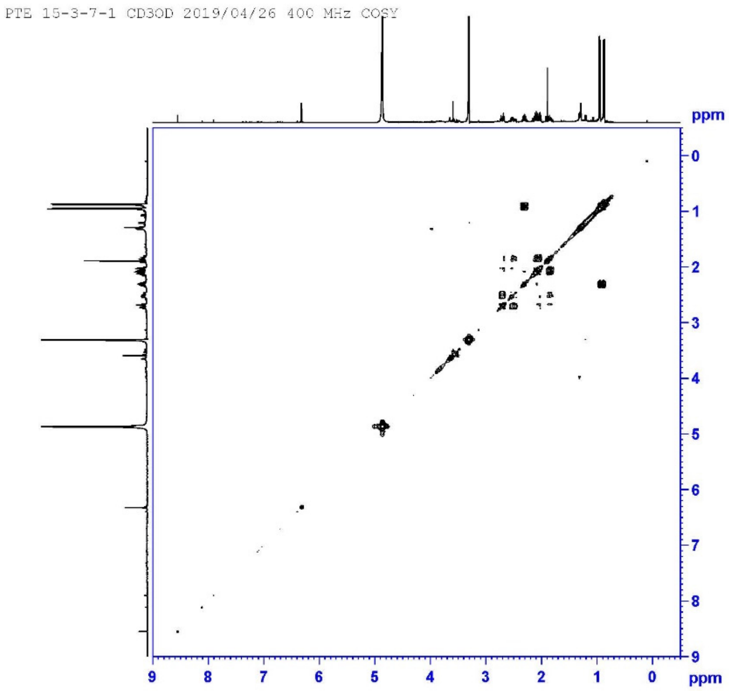

Figure S28. HSQC spectrum of **4** (CD_3OD, 400 MHz).

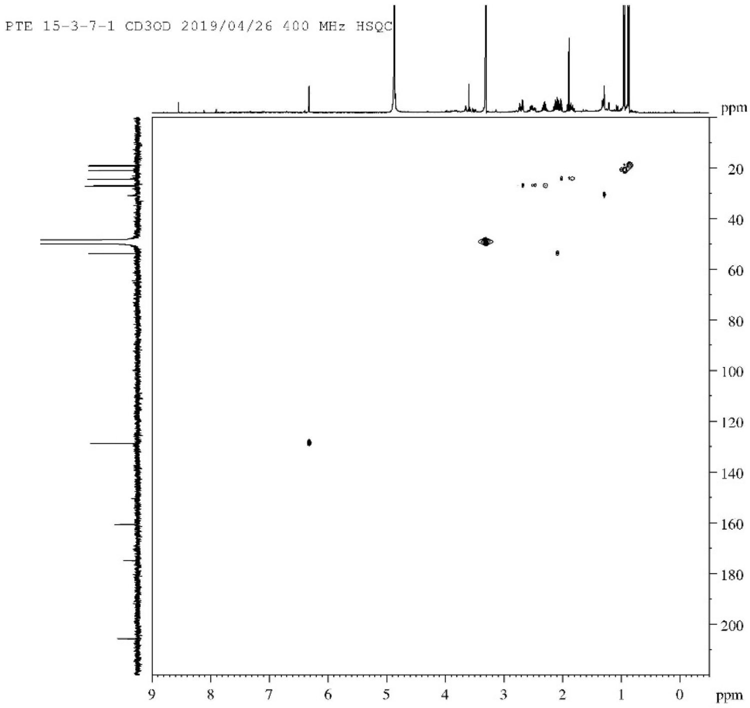

Figure S29. HR-ESI-MS spectrum of **5**.

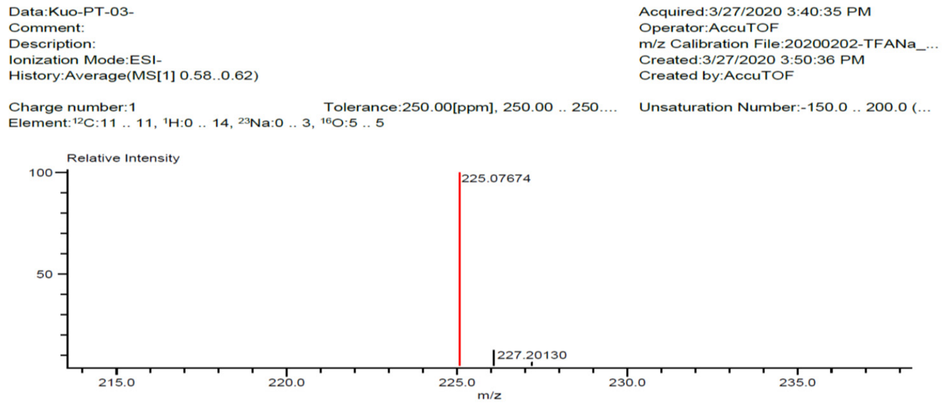

Mass	Intensity	Calc. Mass	Mass Difference [mDa]	Mass Difference [ppm]	Possible Formula
225.07674	28174.82	225.07630	0.44	1.94	$^{12}C_{11}{}^{1}H_{13}{}^{16}O_5$

Figure S30. ^{1}H NMR spectrum of **5** (CD_3OD, 400 MHz).

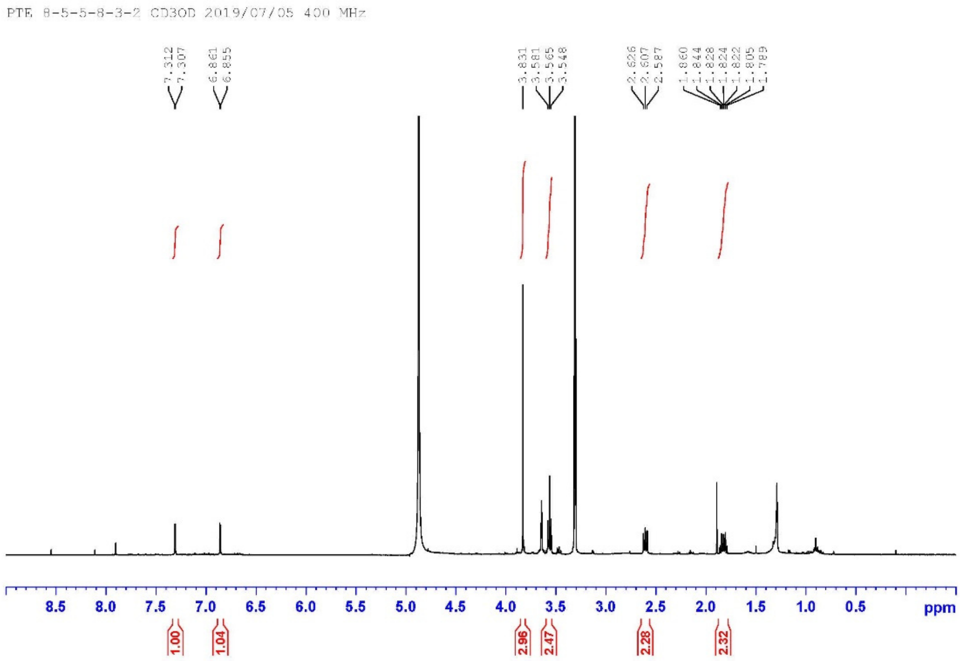

Figure S31. ^{13}C and DEPT NMR spectrum of **5** (CD_3OD, 100 MHz).

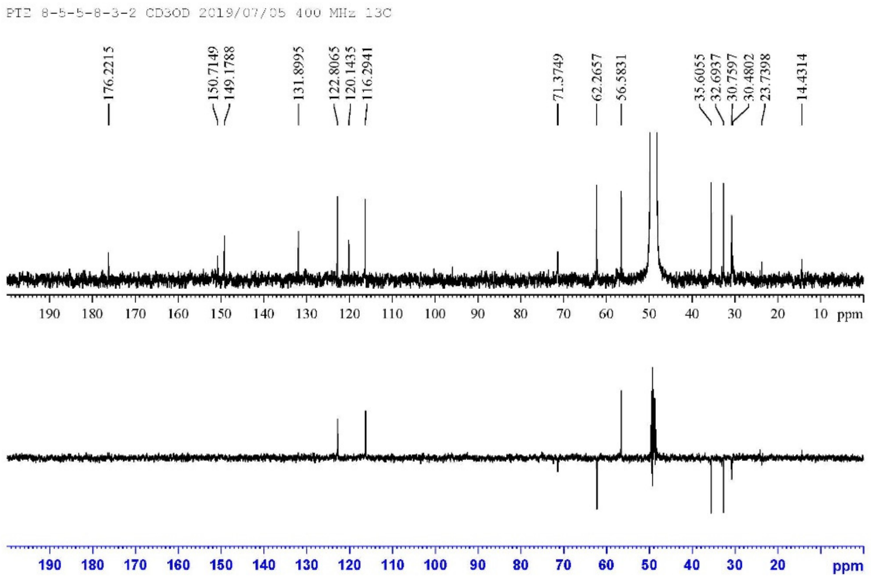

Figure S32. HMBC spectrum of **5** (CD_3OD, 400 MHz).

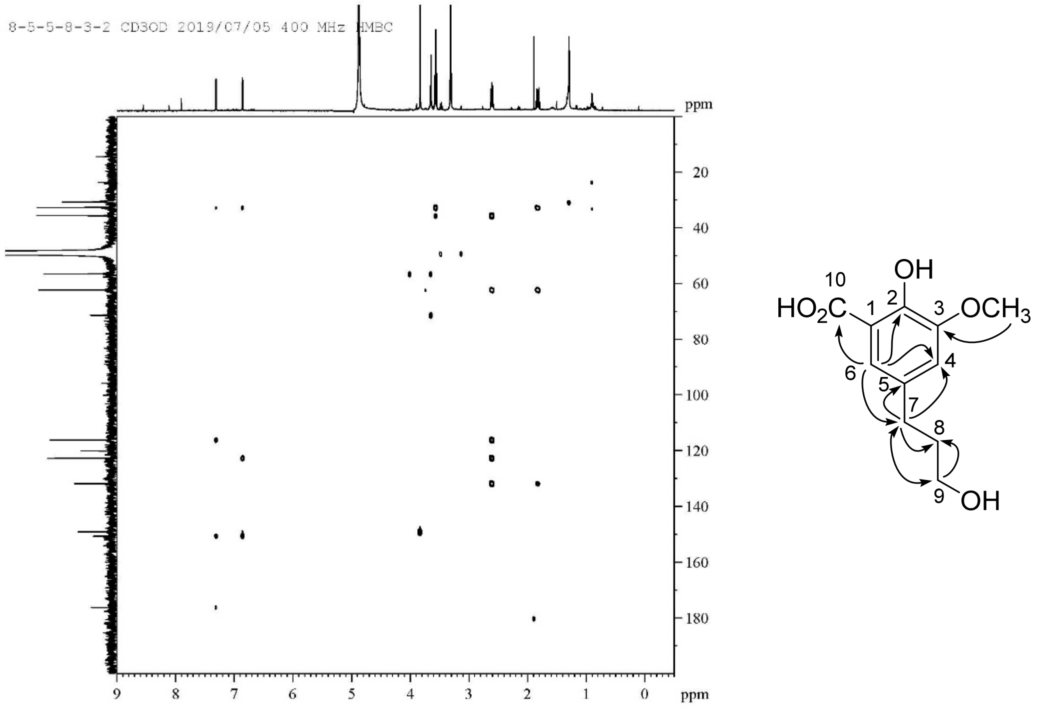

Figure S33. NOESY spectrum of **5** (CD_3OD, 400 MHz).

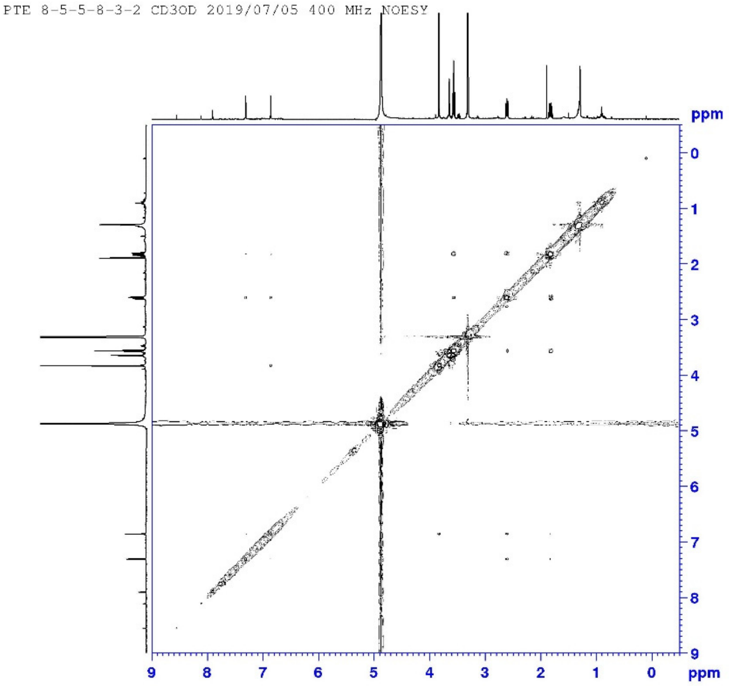

Figure S34. COSY spectrum of **5** (CD_3OD, 400 MHz).

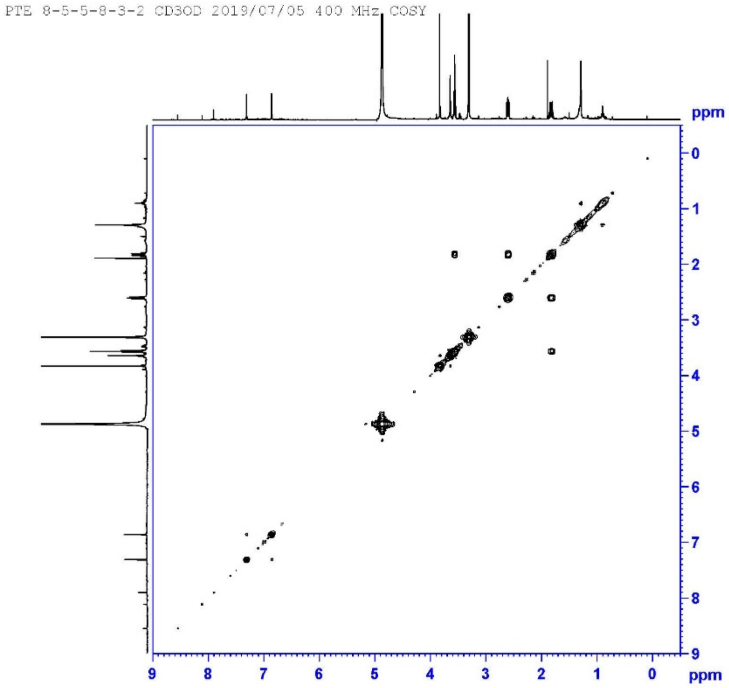

Figure S35. HSQC spectrum of **5** (CD_3OD, 400 MHz).

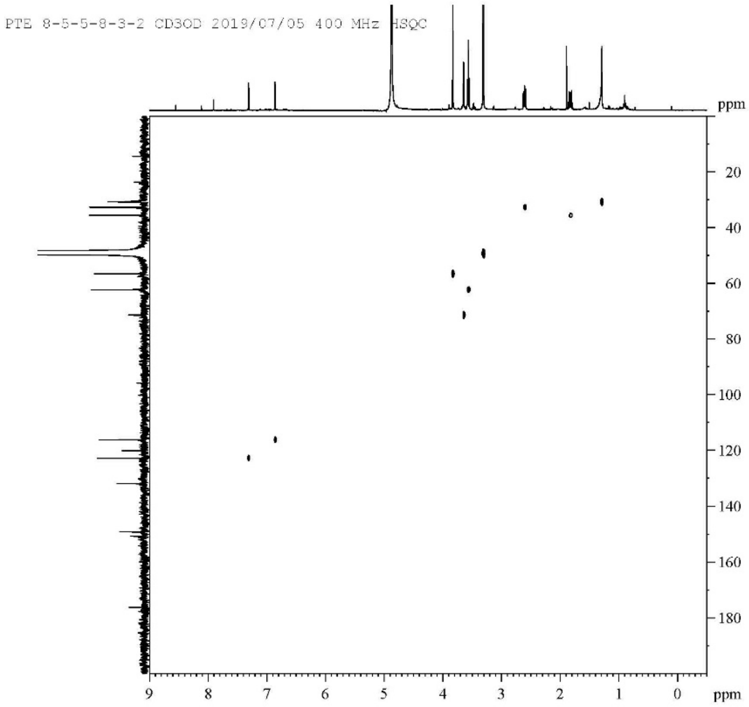

Supplement: Supplementary file 1 [file antioxidants-10-00598-s001.pdf]
